# Supplementary material for: Wound-inducible ANAC071 and ANAC096 transcription factors promote cambial cell formation in incised Arabidopsis flowering stems
Source: Commun Biol. 2021 Mar 19;4:369. doi: 10.1038/s42003-021-01895-8 (PMC7979829; doi:10.1038/s42003-021-01895-8)
Supplement: Supplementary file 2 — Supplementary Information [file 42003_2021_1895_MOESM2_ESM.pdf]

Supplementary information

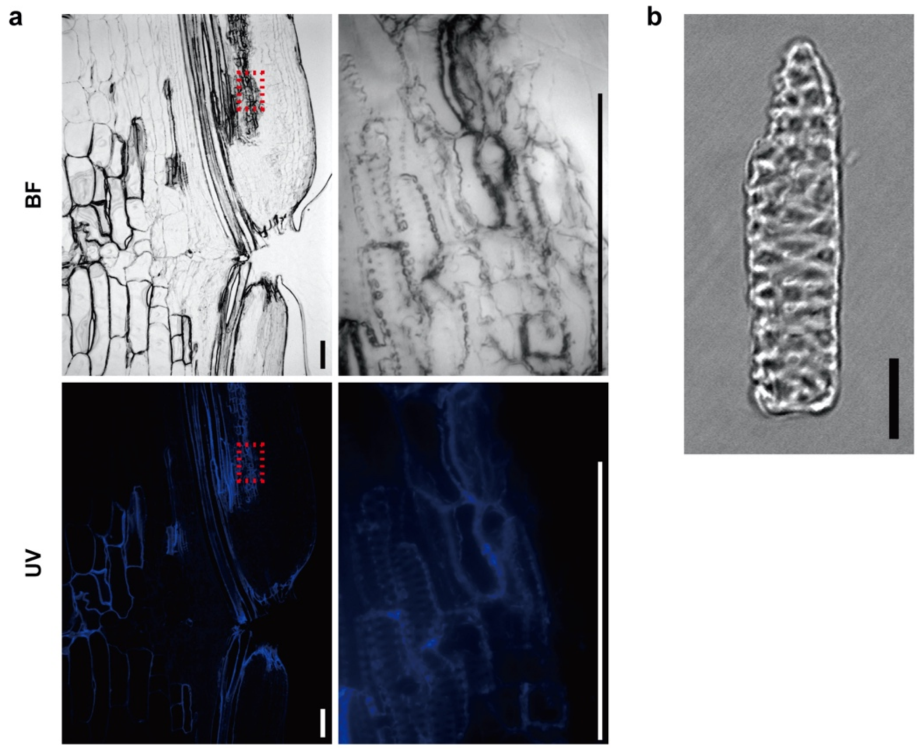

Supplementary Fig. 1 Xylem redifferentiation from wound-induced cambium of intravascular region in incised flowering stem

(**a**) Longisections of incised flowering stem at 7 DAI. Top: bright-field image. Bottom: autofluorescence under UV light. The regions in red dotted-line boxes are shown at higher magnification (right). Scale bars = 100 µm. (**b**) One cell of wound-induced secondary xylem was prepared from xylem maceration of an incised flowering stem at 7 DAI. Scale bar = 10 µm.

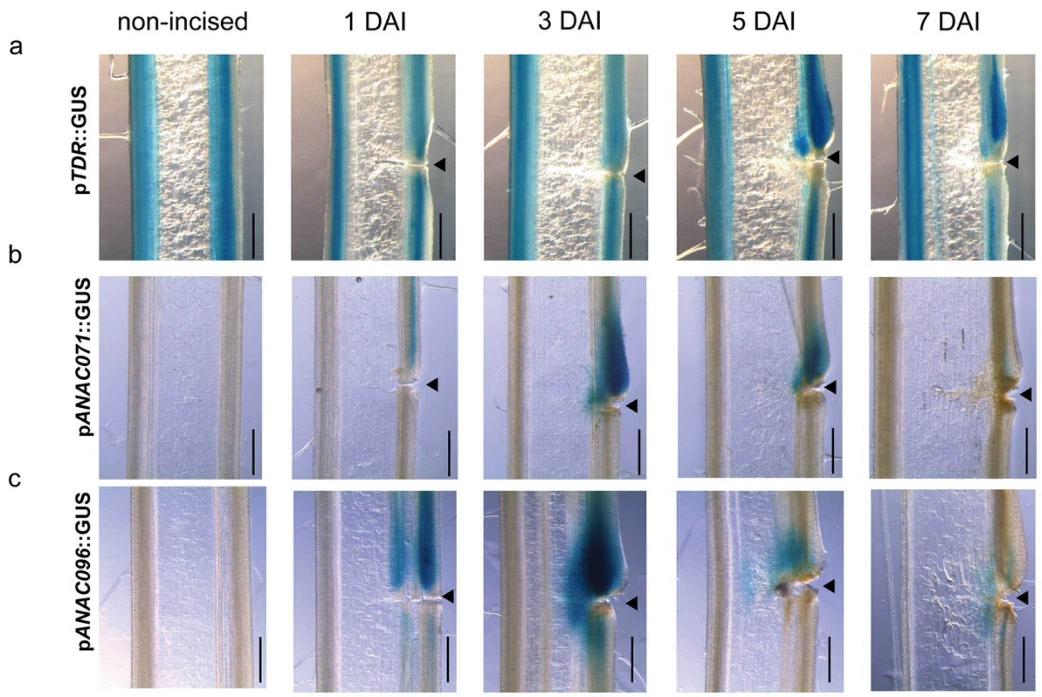

Supplementary Fig. 2 Histochemical analysis of (a) pTDR::GUS, (b) pANAC071::GUS and (c) pANAC096::GUS in non-incised flowering stem and incised flowering stem at 1, 3, 5, 7 DAI. Arrowheads: position of the cut. Scale bars: 500 µm.

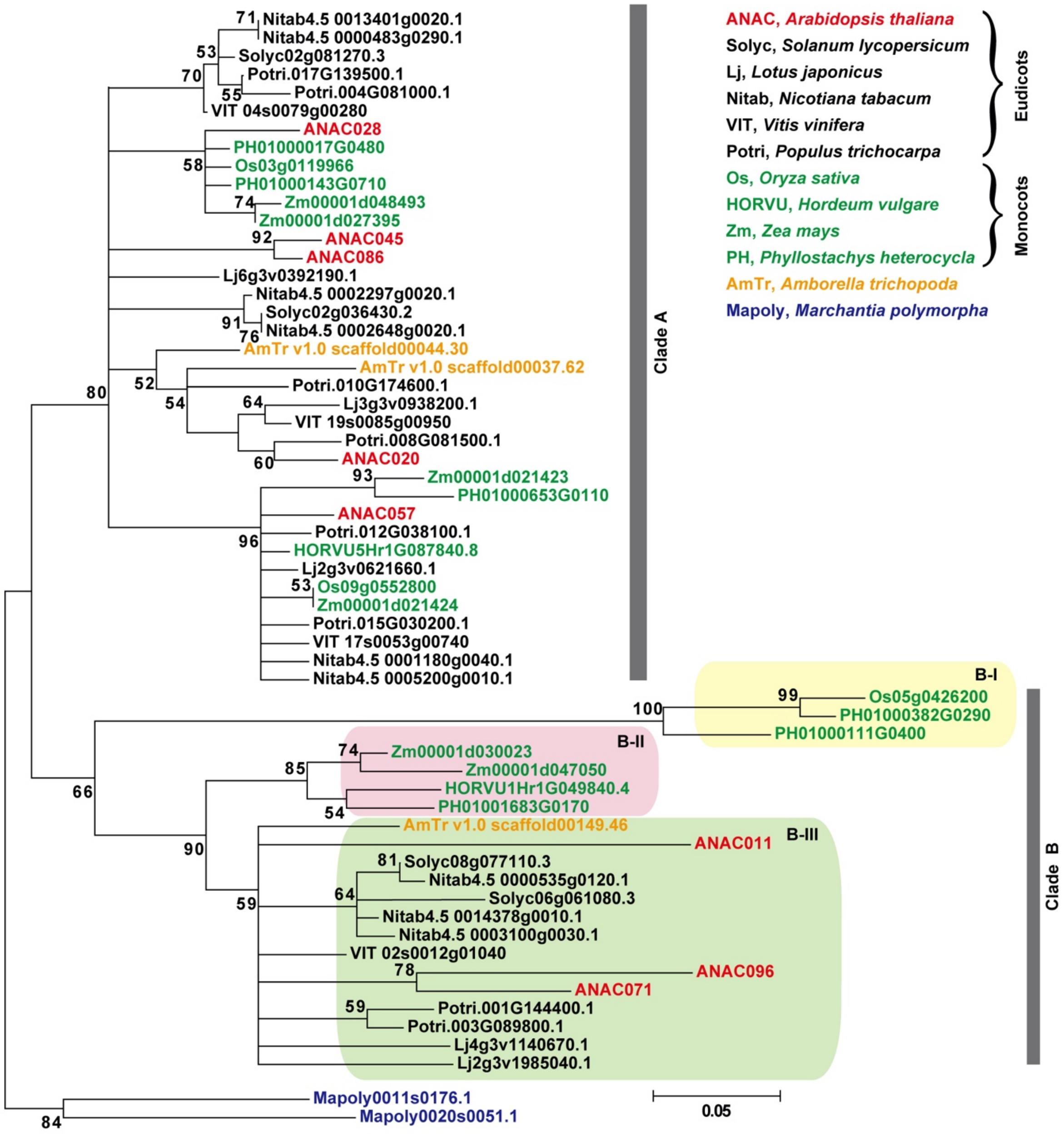

Supplementary Fig. 3 Phylogenetic tree of NAC-type transcription factor

Unrooted neighbor-joining tree obtained using protein sequence alignment of NAC domain in ANAC071 homologues genes. Gene names beginning with words indicate species origin as follows: ANAC (*Arabidopsis thaliana*), Solyc (*Solanum lycopersicum*), Lj (*Lotus japonicas*),

Nitab (*Nicotiana tabacum*), VIT (*Vitis vinifera*), Potri (*Populus trichocarpa*), Os (*Oryza sativa*), HORVU (*Hordeum vulgare*), Zm (*Zea mays*), PH (*Phyllostachys heterocycle*), AmTr (*Amborella trichopoda*), and Mapoly (*Marchantia polymorpha*). The tree was rooted using NAC genes of *Marchantia polymorpha* as an outgroup. Numbers at branch represent the percentage of bootstrap values (1,000 replicates). Multifurcating nodes represent less than 50% of the bootstrap value.

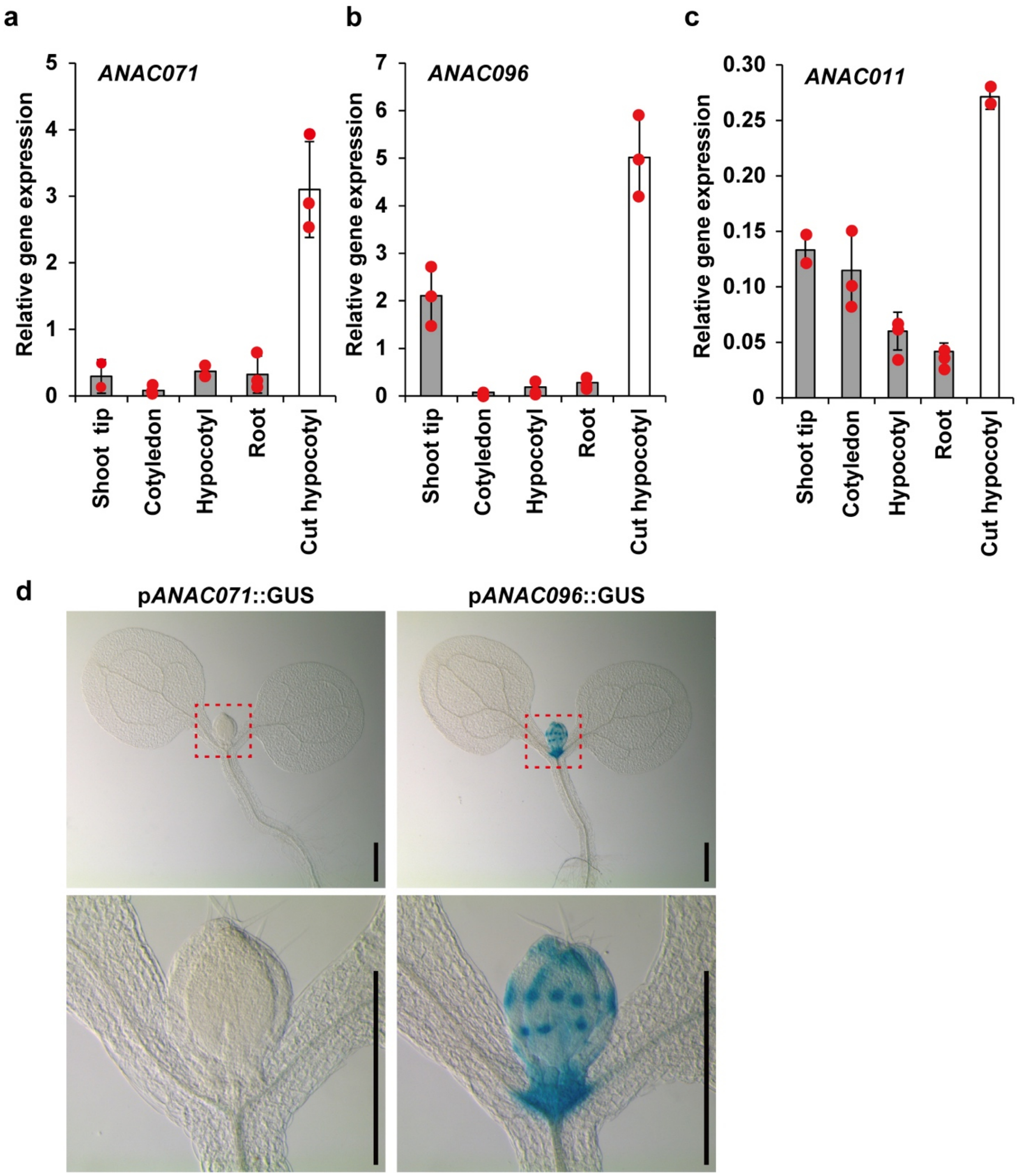

Supplementary Fig. 4 ANAC gene expression in seedlings

(**a**–**c**) Relative gene expression of *ANAC071*, *ANAC096*, and *ANAC011* were analyzed with qRT-PCR. Each organ was separately harvested from 6-day-old seedlings. Hypocotyls were sampled at 1 day after cut in the middle (cut hypocotyl, right bar). (**d**) p*ANAC071*::GUS and

p*ANAC096*::GUS were stained at 6 days after sawing. Bottom, magnified image of shoot tip.

Scale bars = 500 μm.

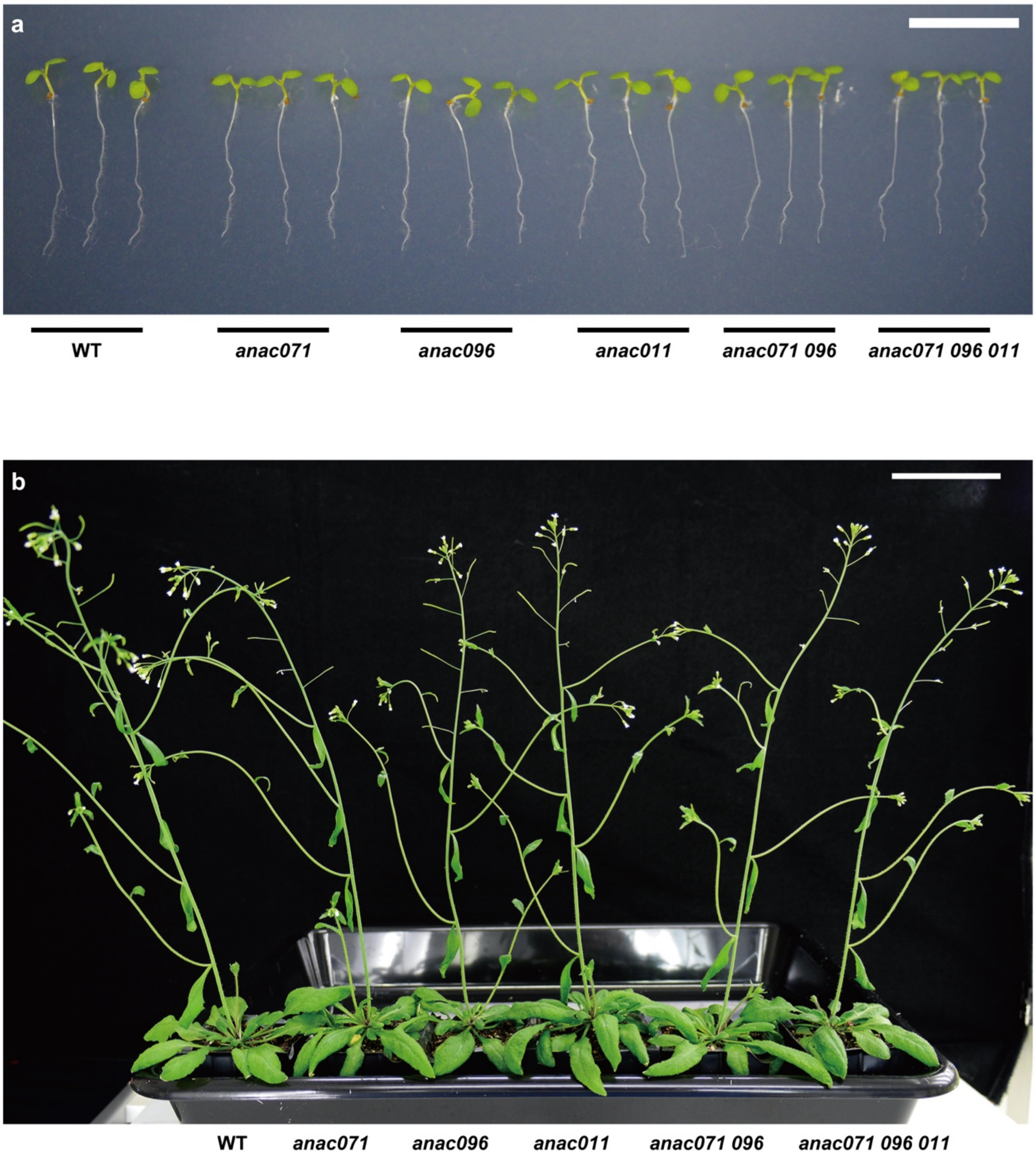

Supplementary Fig. 5 Phenotypes of *anac* mutants

Observation of 6-day-old (**a**) and 5-week-old plants (**b**) in the WT and *anac* mutants. Scale bars = 10 mm, 50 mm.

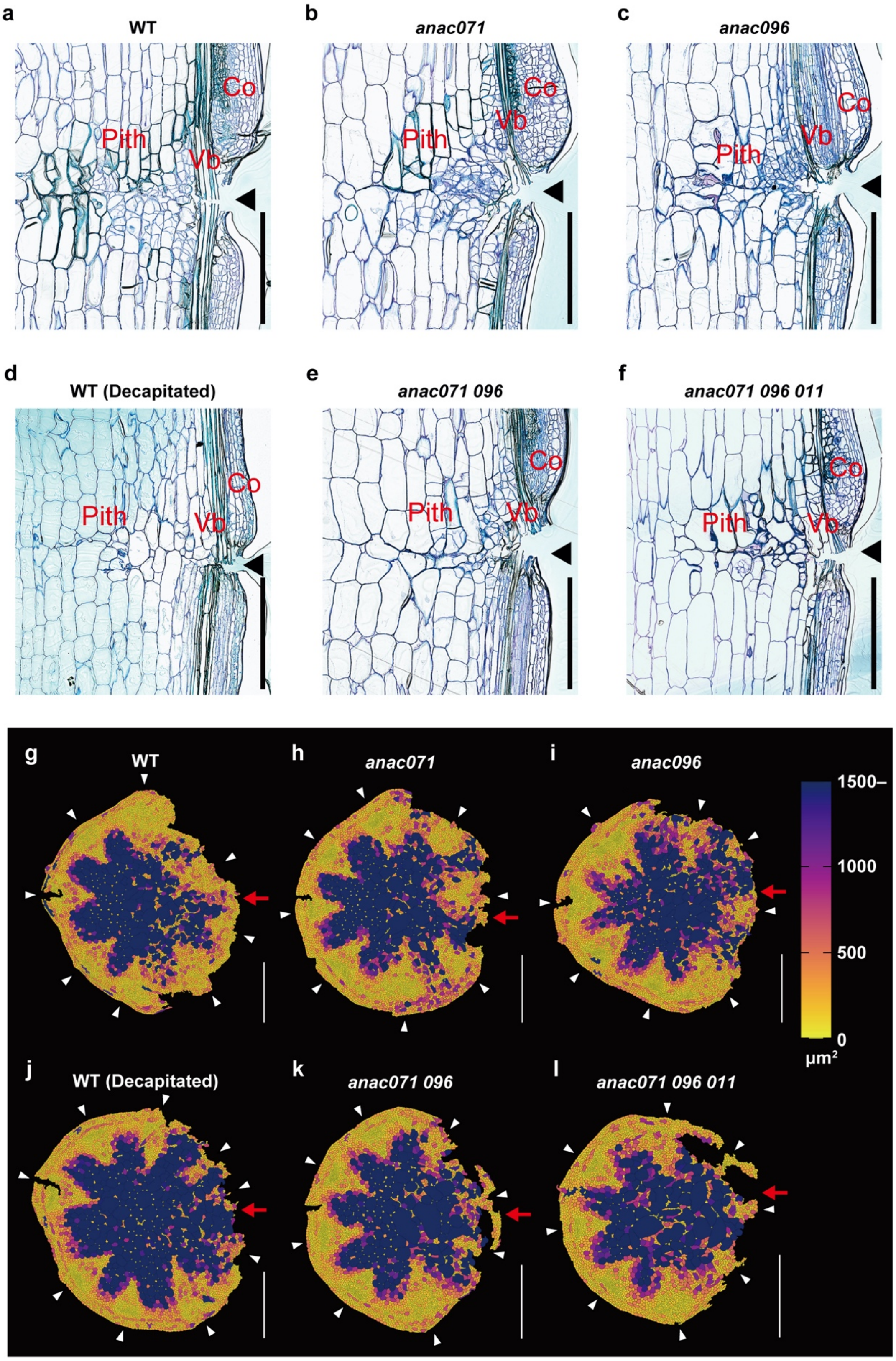

Supplementary Fig. 6 Sections from incised stems of *anac* mutants

(**a**–**f**) Thin longisections were prepared from incised stems at 7 DAI and located between vascular bundles (corresponding to red arrows in g–l). Arrowheads indicate the incised position. Scale bars = 100 μm. cortex, Co; vascular bundle, Vb;.pith, Pith. (**g**–**l**) Thin cross-sections were located in the incised position at 7 DAI. Incised sides are displayed on the right side. Gradational colors were applied to cells according to cell size using the Tissue Cell Segment Movie macro and ROI Color Coder plug-in from Fiji software. White arrowheads indicate the position of the vascular bundle. Scale bars = 500 μm.

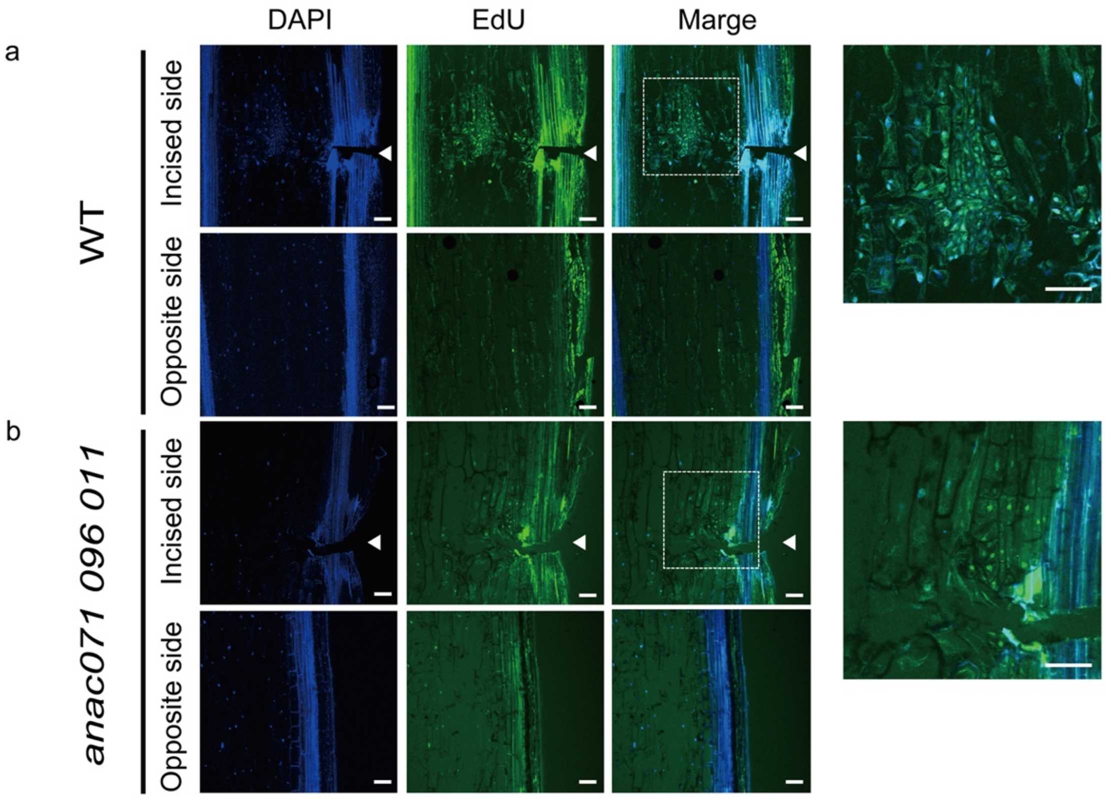

Supplementary Fig. 7 DAPI-staining and EdU-labeling. Cryosections were prepared from incised stem at 5DAI and intact stem of wild-type (a) or *anac071 096 011* mutant (b), respectively. The first and second column provide images taken in the DAPI and the EdU and third column shows a merge of the DAPI and the EdU image. The right column represents a magnified image of the area marked in merge. White arrowheads indicate the position of the cut. Scale bars indicate 100 µm.

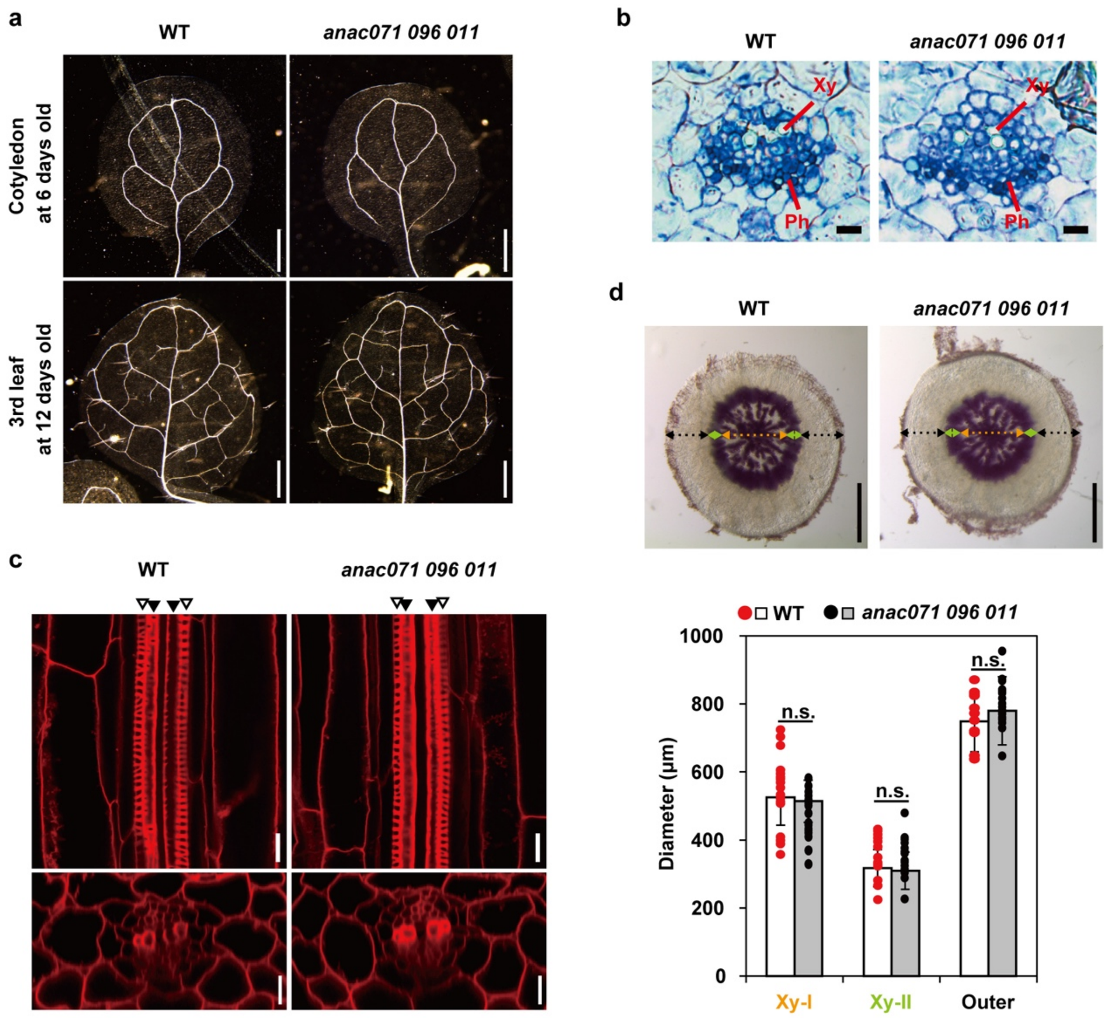

Supplementary Fig. 8 Vascular tissue under ordinary growth conditions

(**a**) Vein patterns in WT and *anac071 096 011* triple mutants are shown in dark-field images. Top; cotyledon from 6-day-old plant, bottom; 3rd leaf from 12-day-old plant. (**b**) Cross-section of vascular tissue at petiole. Xy, xylem; Ph, phloem. Scale bars = 10 µm. (**c**) Xylem vessels of mature roots from 6-day-old plants. Roots were stained with modified pseudo-Schiff propidium iodide and observed by confocal laser microscopy (top). Optical cross-sections were generated from 104 z-stack images with 0.5 µm intervals (bottom). Open

triangle, protoxylem; closed triangle, metaxylem. Scale bars = 10 μm. (**d**) Comparison of secondary xylem formation at hypocotyl after bolting. Freehand cross-sections were stained for lignin with phloroglucinol reagent (top). Orange, light green, and black regions indicate xylem-I (Xy-I, containing parenchyma cells), xylem-II (Xy-II, composed of xylem fibers with lignified cells) and outer (tissues excluding xylem), respectively. Scale bars = 500 μm. Diameters of each region were measured from 20 plants (bottom, means±SD). White bars, WT; gray bars, *anac071 096 011*. n.s., not significant; ***P* < 0.05 (Welch's t test).

Xylem cells unconnected to other xylem strands were frequently observed in the vascular pattern because of the altered perception or transport of auxin, as in the *mp*, *cvp* or *fkd* mutants[1-3]. The *anac071 096 011* triple mutant showed a normal vein pattern, which consisted of a xylem strand connected at the cotyledon and 3rd leaf (**a**). From the cross-section at the petiole and optical section at the root, no difference in size and constitution of vascular tissue was observed between the WT and the *anac071 096 011* triple mutant (**b**, **c**). The diameter of xylem tissue was not significantly different between the WT and the *anac071 096 011* triple mutant in the hypocotyl at the stage after bolting, indicating that cambial activities were the same during secondary growth (**d**).

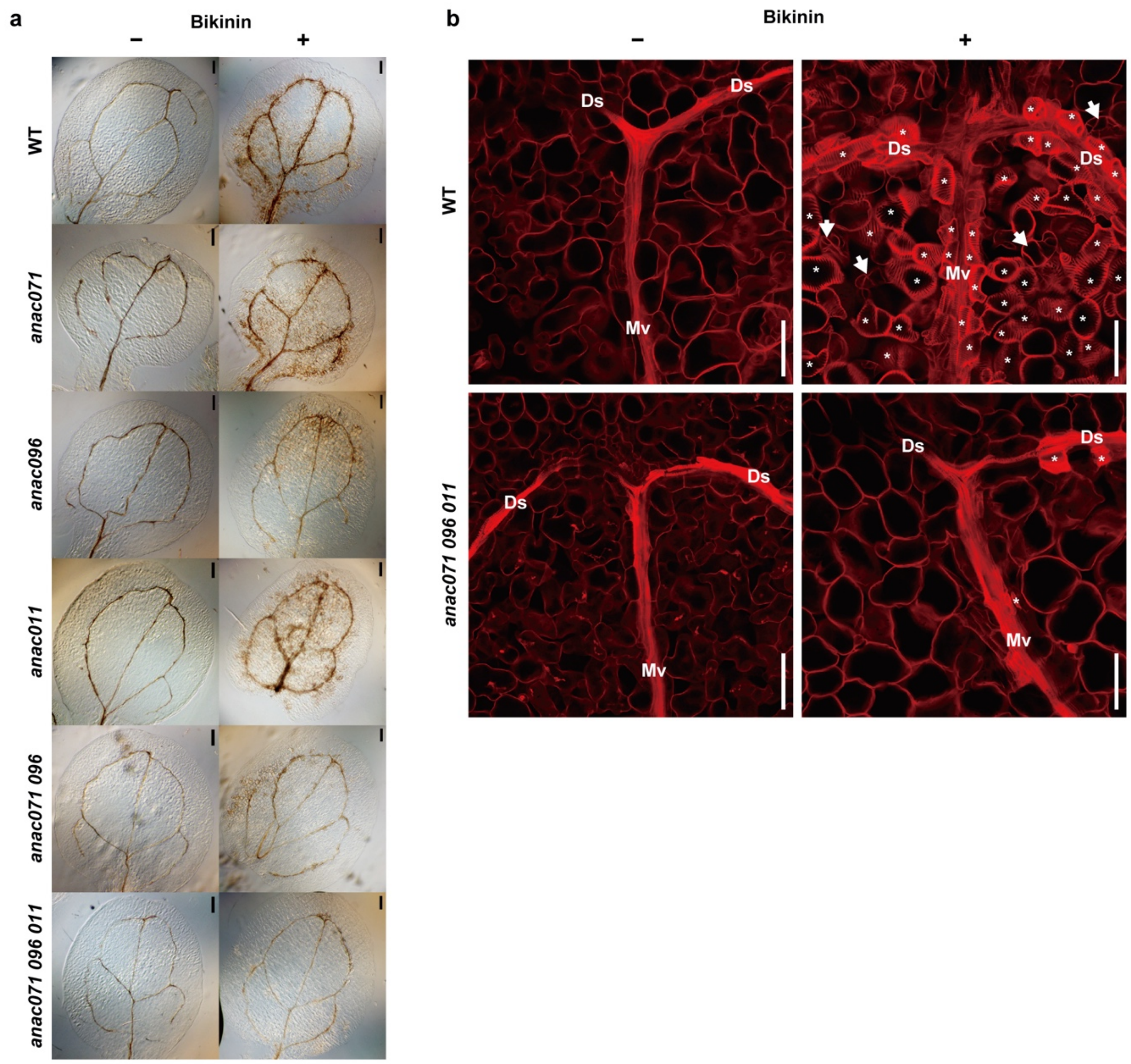

Supplementary Fig. 9 VISUAL assay in *anac* mutants

The VISUAL assay was performed with (+) or without (−) bikinin at 4 DOC. (**a**) Bright-field images were acquired from cleared cotyledons in the WT and the *anac071*, *anac096*, *anac011*, *anac071 096* double and *anac071 096 011* triple mutants. Scale bars = 200 µm. (**b**) Cotyledons of the WT and *anac071 096 011* triple mutant were stained using the modified pseudo-Schiff propidium iodide method. Optical sections are displayed at the junction

between the midvein and distal secondary veins. Asterisks and arrows indicate ectopic xylem and ectopic phloem cells, respectively. Midvein, Mv; distal secondary vein, Ds. Scale bar = 100 μm.

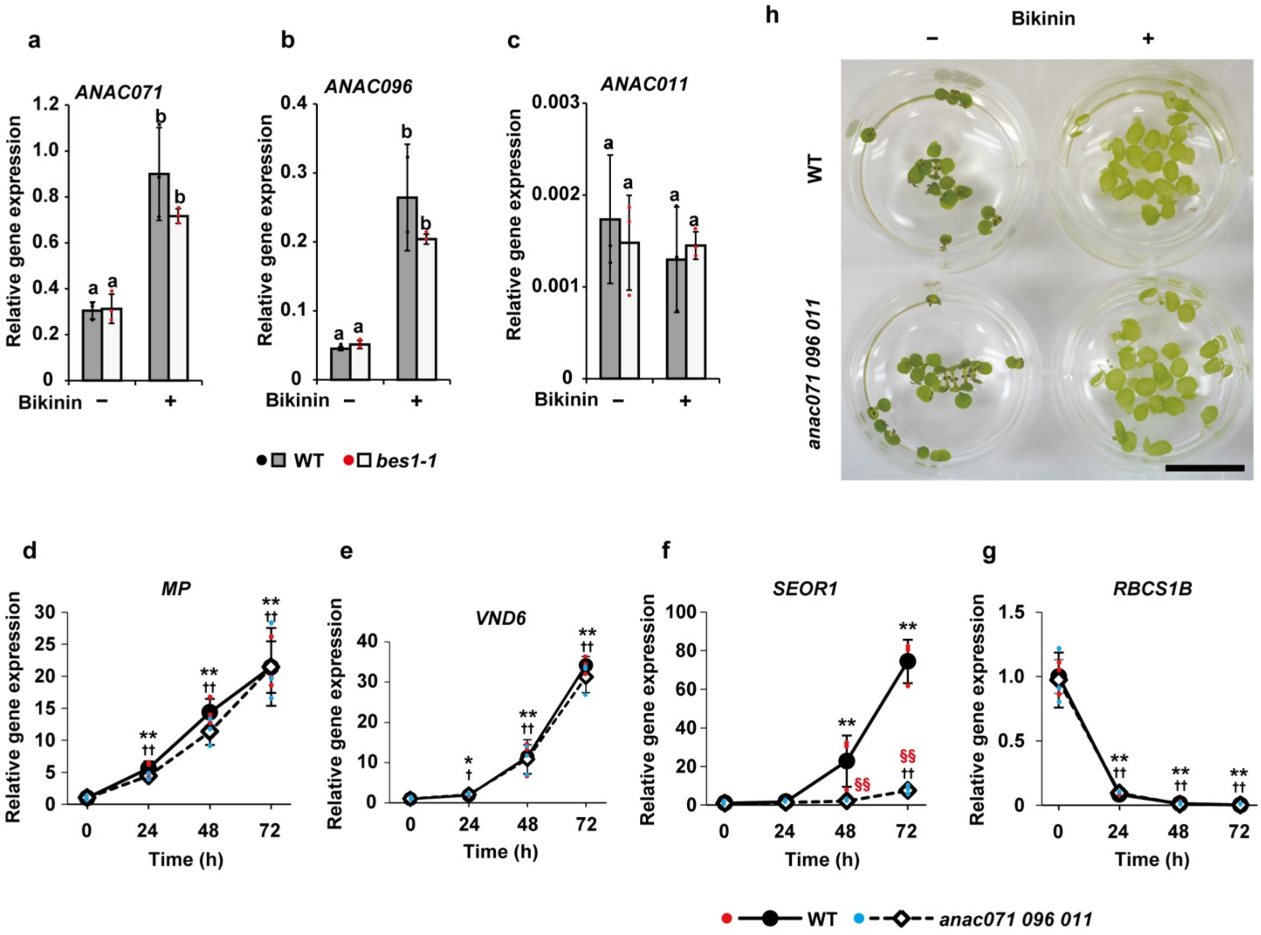

Supplementary Fig. 10 Gene expression analysis in VISUAL assay

(**a**–**c**) ANAC gene expression in WT (gray bars) and *bes1-2* (white bars) at 2 DOC. −, control; +, application of bikinin. The relative expression levels were analyzed using qRT-PCR and normalized to ACT2 expression (means±SD from triplicate batch experiments). (**d**–**g**) Relative gene expression of *MP*, *VND6*, *SEOR1* and *RBCS1B* was analyzed by the VISUAL assay. The experimental conditions were as described in Fig. 6e and f. Relative gene expression was calculated when WT 0h was set to 1. (**h**) Macroscopic views of WT (top) and

anac071 096 011 triple mutant (bottom). Scale bars = 10 mm.

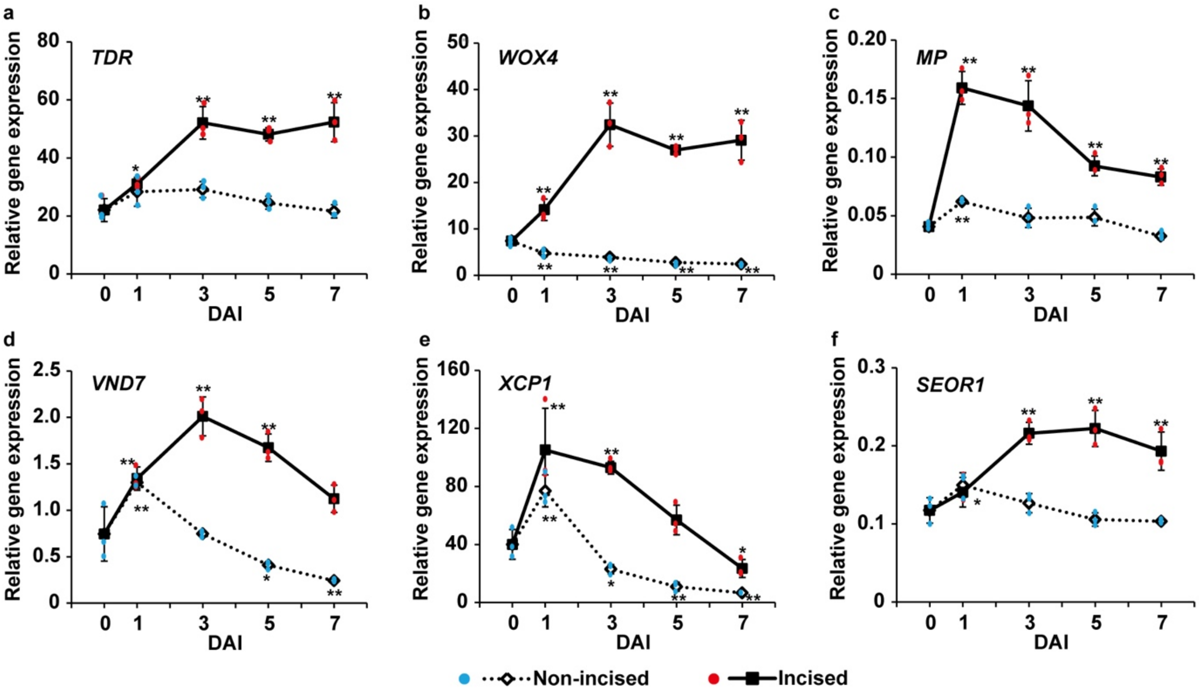

Supplementary Fig. 11 Gene expression analysis during 1 to 7 days after incision

Relative gene expression of *TDR*, *WOX4*, *MP*, *VND7*, *XCP1* and *SEOR1* was analyzed in flowering stems at 1–7 days after incision (DAI). The experimental conditions were as described in Fig. 2a–d. Asterisks indicate statistically significant differences compared with day 0 (**P < 0.01; Dunnett's test).

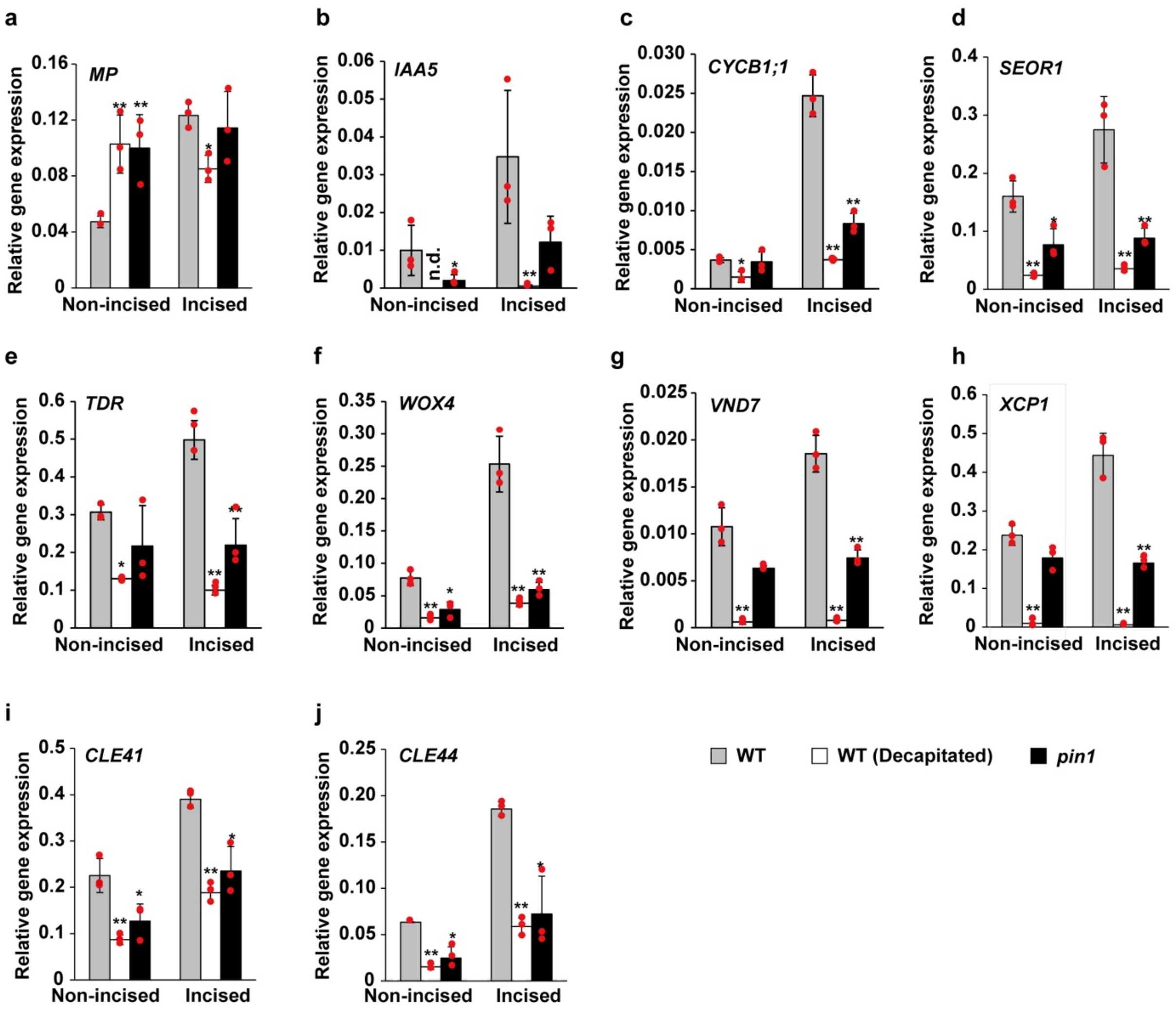

Supplementary Fig. 12 Gene expression analysis in decapitated WT and *pin1* mutant

The relative expression levels of *MP*, *IAA5*, *CYCB1;1*, *SEOR1*, *TDR*, *WOX4*, *VND7*, *XCP1*, *CLE41* and *CLE44* in flowering stems at 3 DAI were analyzed. The experimental conditions were as described in Fig. 2e–g. n.d., not detected.

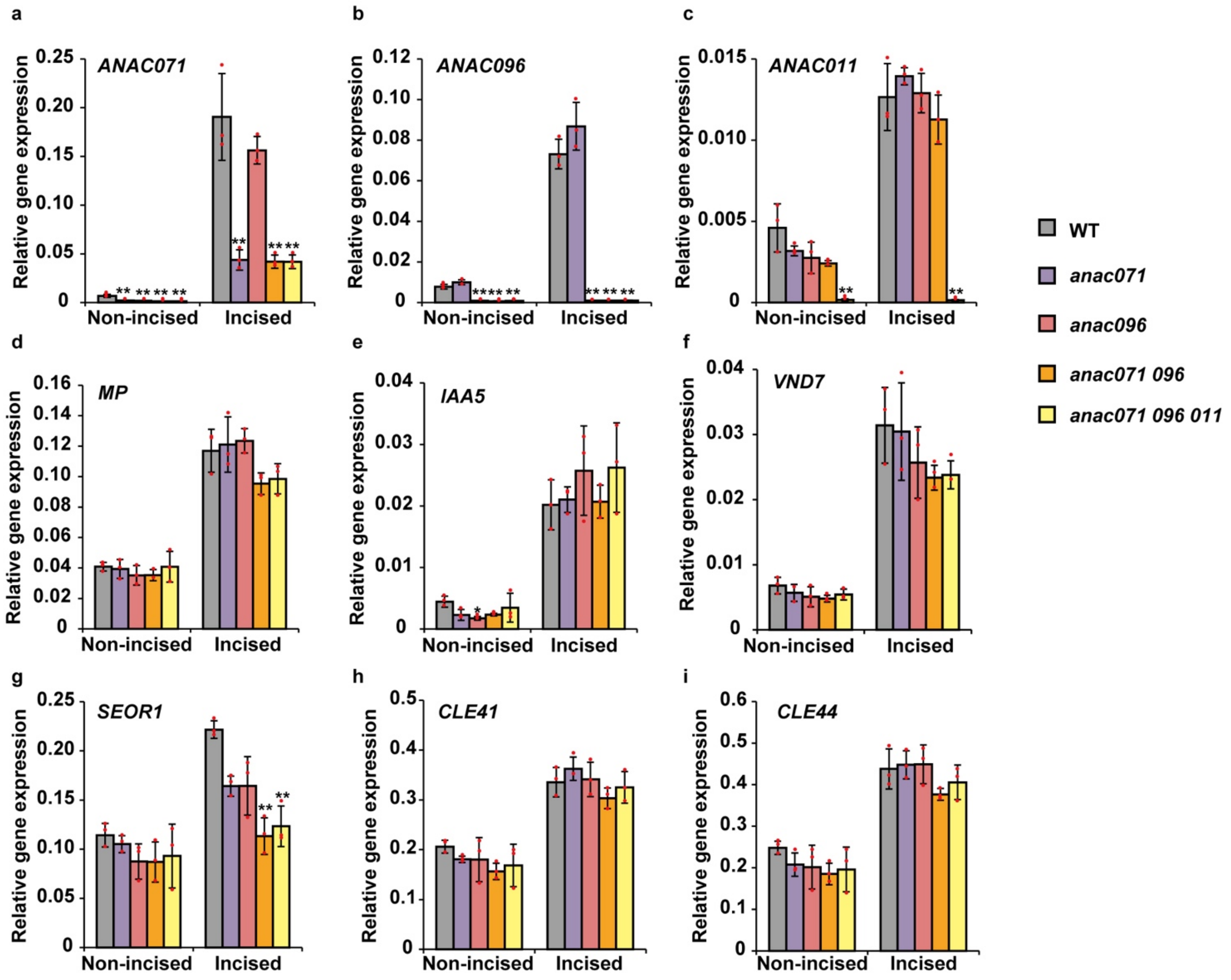

Supplementary Fig. 13 Gene expression analysis in *anac* mutants.

Relative gene expression of *ANAC071*, *ANAC096*, *ANAC011*, *MP*, *IAA5*, *VND7*, *XCP1*, *SEOR1*, *CLE41*, and *CLE44* was analyzed in flowering stems at 3 DAI. The experimental conditions were as described in Fig. 7a. Asterisks indicate statistically significant differences compared with the WT (***P* < 0.01; Dunnett's test).

Supplementary references

1. Przemeck, G. K. H., Mattsson, J., Hardtke, C. S., Sung, Z. R. & Berleth, T. Studies on the role of the *Arabidopsis* gene *MONOPTEROS* in vascular development and plant cell axialization. *Planta* **200**, 229-237 (1996).

2. Carland, F. M. et al. Genetic regulation of vascular tissue patterning in *Arabidopsis*. *Plant Cell* **11**, 2123-2137 (1999).

3. Steynen, Q. J. & Schultz, E. A. The FORKED genes are essential for distal vein meeting in *Arabidopsis*. *Development* **130**, 4695-4708 (2003).

Table S1. List of primers used in this study

For quantitative RT-PCR

AGI number	Description	Sequence	
AT3G18780	ACT2	Fwd	5’-CTGGATCGGTGGTTCCATTC-3’
		Rev	5’-CCTGGACCTGCCTCATCATAC-3’
AT4G17980	ANAC071	Fwd	5'-CCTCTCCTTGTCGCGATGAA-3'
		Rev	5'-ATGCTTGAAGAGTCGTTTGTAGTAGAAG-3'
AT5G46590	ANAC096	Fwd	5'-ACATGGAGTTTCCAGCGAGT-3'
		Rev	5'-ACCGGTTTGATCGTATCCAT-3'
AT1G32510	ANAC011	Fwd	5'-CCGAATTCGAATATCCGCTAGA-3'
		Rev	5'-TGTTCATATGAGGCTCTGGATG-3'
AT5G61480	TDR	Fwd	5'-ACATCGGACATTGCGAGAAG-3'
		Rev	5'-GATCTACGTCGGCGATTGAC-3'
At1g71930	VND7	Fwd	5'-CACCATGCATCAATATGGCAACATTGAG-3'
		Rev	5'-TAGTGTTCTCCAATCCACACAGTT-3'
AT1G46480	WOX4	Fwd	5'-AGGTGGAACCCGACTCAAG-3'
		Rev	5'-CGTACTTACCGAGTTGCAATGTG-3'
AT4G37490	CYCB1;1	Fwd	5’-GAACTGCAGCTTGTTGGTCTCA-3’
		Rev	5’-CACCTGTGGTGGCCAAATTT-3’
AT1G19850	MP	Fwd	5'-GCAGACTCACAGGCCTTCTC-3'
		Rev	5'-TGCCGCAGACTACAATCATC-3'
AT4G35350	XCP1	Fwd	5'-GCGGTTGGATATGGTTCATC-3'
		Rev	5'-CCTCTGGTTTACCAGTGTTTCTC-3'
AT3G01680	SEOR1	Fwd	5'-CAGATCAGGCGTATCAGTGAAG-3'
		Rev	5'-CTCGAGCCTAGTCCAGAAGAAC-3'
AT3G54890	LHCA1	Fwd	5'-CGTGTACCCTTCGCTTCTCT-3'
		Rev	5'-ATCCAGTGAGCAGCCATTCT-3'
AT1G29910<br>AT1G29920<br>AT1G29930	LHCB1	Fwd	5'-GGAGACTACGGATGGGACAC-3'
		Rev	5'-CCCACCTGCTGTGGATAACT-3'
AT3G24770	CLE41	Fwd	5’-CCCATGACTCGTCATCAGTC-3’
		Rev	5’-TGGACGTAGATCCATTGTTGA-3’
AT4G13195	CLE44	Fwd	5’-CAGCTCACCCTAGACGCAGT-3’
		Rev	5’-TGGACCACTTGGAACCTCA-3’
AT1G15580	IAA5	Fwd	5’-CCGGCGAAAAAGAGTCAAGT-3’
		Rev	5’-GACTGTTCTTTCTCCGGTACGAA-3’

For genotyping

AGI number	Description	Sequence	
AT4G17980	ANAC071	Fwd	5'-CATCGCATGTTATAGCTGGATAC-3'
		Rev	5'-CTTCTTCTTCCAAGTTCCAAGAC-3'
	anac071	Fwd	5'-CATCGCATGTTATAGCTGGATAC-3'
		LBb1.3	5'-ATTTTGCCGATTTCGGAAC-3'
AT5G46590	ANAC096	Fwd	5'-GTCTCGAGATTGAGCTTGAAGTC-3'
		Rev	5'-GGCATTGTTTCATCGCGACTAG-3'
	anac096	Rev	5'-GGCATTGTTTCATCGCGACTAG-3'
		LBb1.3	5'-ATTTTGCCGATTTCGGAAC-3'
AT1G32510	ANAC011	Fwd	5'-GTAGGTTATTACCTGCACAGAAG-3'
		Rev	5'-CGTGTGTTACTTGCATCACAAG-3'
	anac011	Fwd	5'-GTAGGTTATTACCTGCACAGAAG-3'
		o3144	5'-GTGGATTGATGTGATATCTCC-3'
AT1G73590	PIN1	Fwd	5'-CAGCTTTGCCGCAAGG-3'
		Rev	5'-CTCTTCATAGACCCAAGAGAATG-3'
	pin1	Rev	5'-CTCTTCATAGACCCAAGAGAATG-3'
		LBb1.3	5'-ATTTTGCCGATTTCGGAAC-3'
