# Supplementary material for: Wound-inducible ANAC071 and ANAC096 transcription factors promote cambial cell formation in incised Arabidopsis flowering stems
Source: Commun Biol. 2021 Mar 19;4:369. doi: 10.1038/s42003-021-01895-8 (PMC7979829; doi:10.1038/s42003-021-01895-8)
Supplement: Supplementary file 3 — Description of Additional Supplementary Files [file 42003_2021_1895_MOESM3_ESM.pdf]

Description of Additional Supplementary Files

**File Name:** Supplementary Data 1
**Description:** All section images for Fig. 4b and all leaf images for Fig. 5b.
Source 1: All section images for Fig. 4b, WT
Source 2: All section images for Fig. 4b, WT(decapitated)
Source 3: All section images for Fig. 4b, *anac071*
Source 4: All section images for Fig. 4b, *anac096*
Source 5: All section images for Fig. 4b, *anac071 096*
Source 6: All section images for Fig. 4b, *anac071 096 011*
Source 7-8: All leaf images for Fig. 5b, WT
Source 9-10: All leaf images for Fig. 5b, *anac071*
Source 11-12: All leaf images for Fig. 5b, *anac096*
Source 13-14: All leaf images for Fig. 5b, *anac011*
Source 15-16: All leaf images for Fig. 5b, *anac071 096*
Source 17-18: All leaf images for Fig. 5b, *anac071 096 011*

**File Name:** Supplementary Data 2
**Description:** The source data used to generate graphs in main figures.
Source 1: Fig.2b-d;Gene expression of ANAC071, ANAC096 and ANAC011 in flowering stems at 1–7 days after incision
Source 2: Fig.2e-d;Gene expression levels of ANAC071, ANAC096, and ANAC011 in flowering stems at 3 DAI.
Source 3: Fig.4b-c;Number of cell layer in secondary xylem and the ratio of cells with secondary cell wall.
Source 4: Fig.5b;The percentage of ectopic xylem area in WT and anac mutants.
Source 5: Fig.6a-c;Gene expression of ANAC under VISUAL assay.
Source 6: Fig.6e-f;Gene expression of TDR/PXY and XCP1 under VISUAL assay.

**File name:** Supplementary Movie 1
**Description:** Time-lapse movie of incised flowering stem.
Movie of incised flowering stem for 7 days at a time-lapse of 30 minutes was captured using a camera (LUMIX, Panasonic) and edited using the free software AviUtl..
