# Supplementary material for: Wound-inducible ANAC071 and ANAC096 transcription factors promote cambial cell formation in incised Arabidopsis flowering stems
Source: Commun Biol. 2021 Mar 19;4:369. doi: 10.1038/s42003-021-01895-8 (PMC7979829; doi:10.1038/s42003-021-01895-8)
Supplement: Supplementary file 5 — Supplementary Data 1 [file 42003_2021_1895_MOESM5_ESM.zip › SupplementaryDataset1/Description.pdf]

File Name: Supplementary Data 1
Description: All section images for Fig. 4b and all leaf images for Fig. 5b.
Source 1: All section images for Fig. 4b, WT
Source 2: All section images for Fig. 4b, WT(decapitated)
Source 3: All section images for Fig. 4b, *anac071*
Source 4: All section images for Fig. 4b, *anac096*
Source 5: All section images for Fig. 4b, *anac071 096*
Source 6: All section images for Fig. 4b, *anac071 096 011*
Source 7-8: All leaf images for Fig. 5b, WT
Source 9-10: All leaf images for Fig. 5b, *anac071*
Source 11-12: All leaf images for Fig. 5b, *anac096*
Source 13-14: All leaf images for Fig. 5b, *anac011*
Source 15-16: All leaf images for Fig. 5b, *anac071 096*
Source 17-18: All leaf images for Fig. 5b, *anac071 096 011*
