# Supplementary figures and images for: Wound-inducible ANAC071 and ANAC096 transcription factors promote cambial cell formation in incised Arabidopsis flowering stems
Source: Commun Biol. 2021 Mar 19;4:369. doi: 10.1038/s42003-021-01895-8 (PMC7979829; doi:10.1038/s42003-021-01895-8)

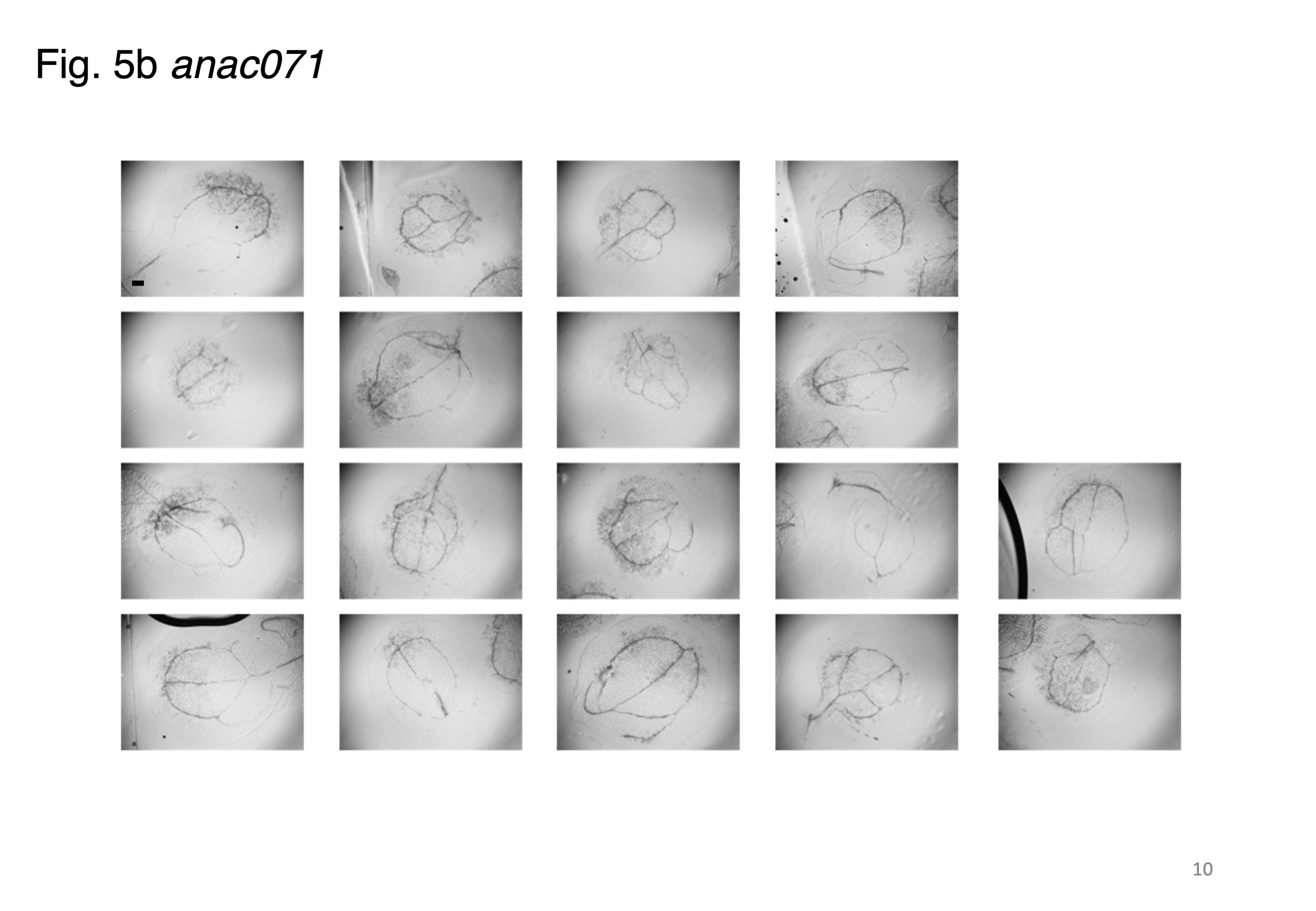

Supplement: Supplementary file 5 — Supplementary Data 1 [file 42003_2021_1895_MOESM5_ESM.zip › SupplementaryDataset1/10.jpeg]

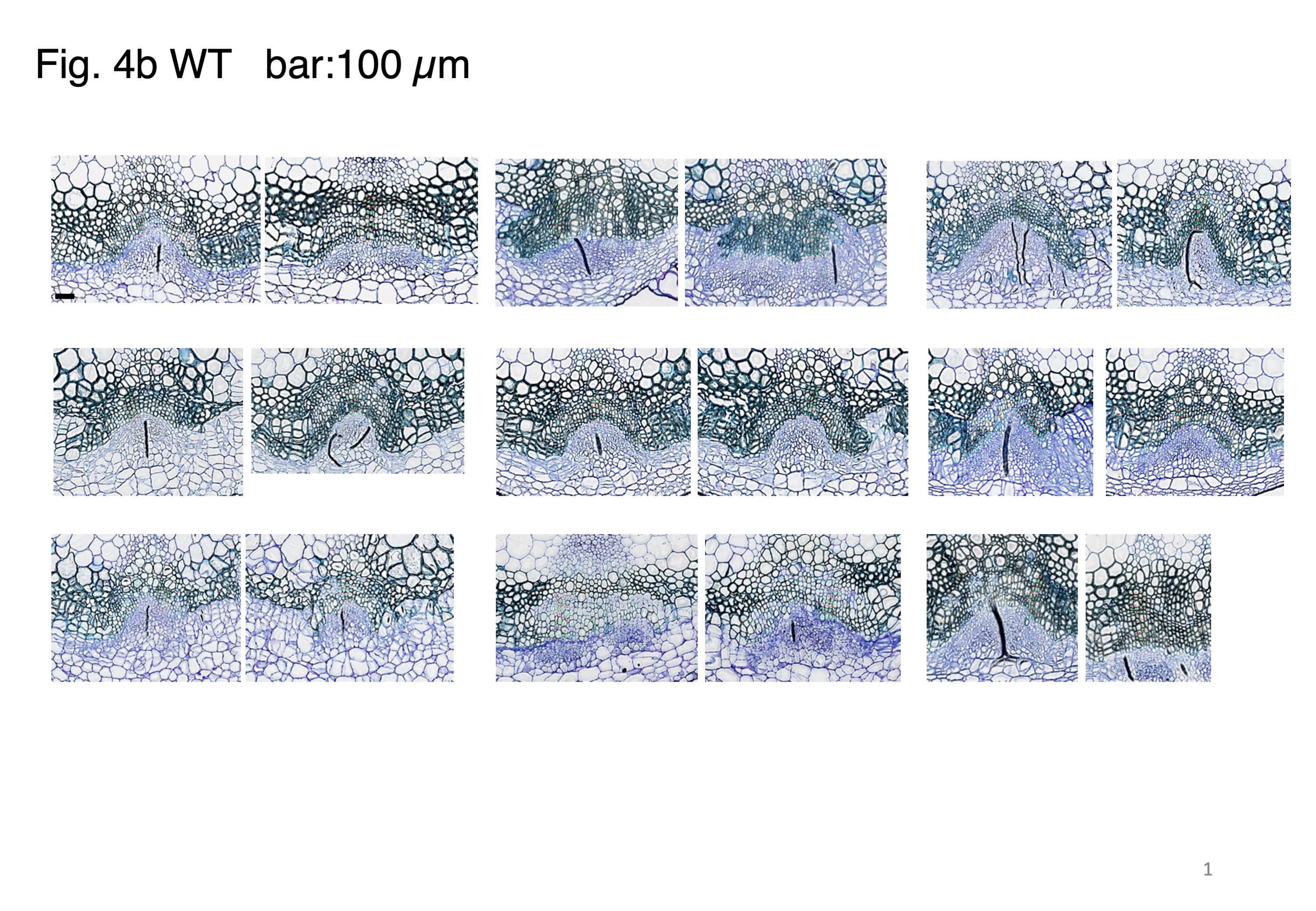

Supplement: Supplementary file 5 — Supplementary Data 1 [file 42003_2021_1895_MOESM5_ESM.zip › SupplementaryDataset1/1.jpeg]

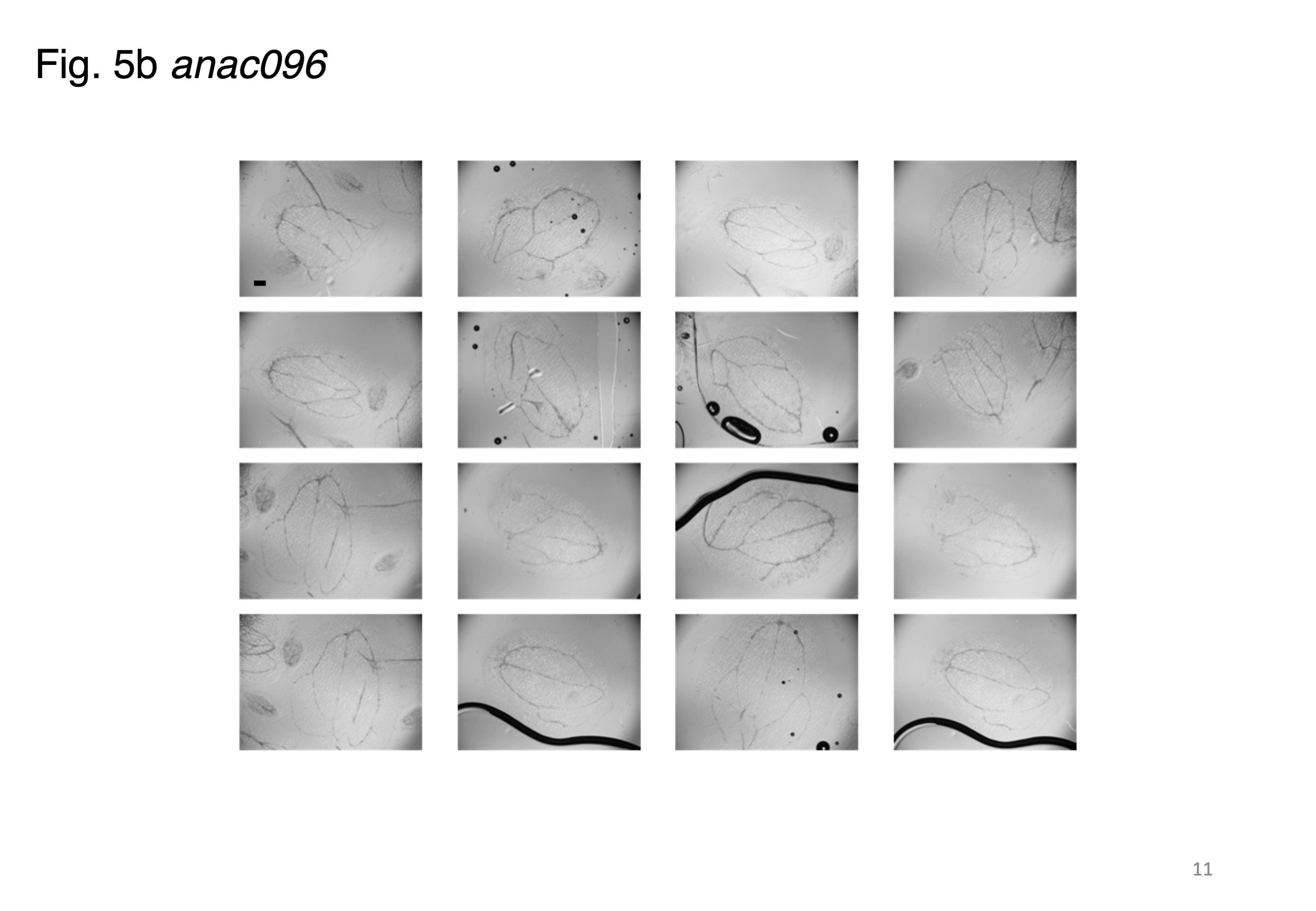

Supplement: Supplementary file 5 — Supplementary Data 1 [file 42003_2021_1895_MOESM5_ESM.zip › SupplementaryDataset1/11.jpeg]

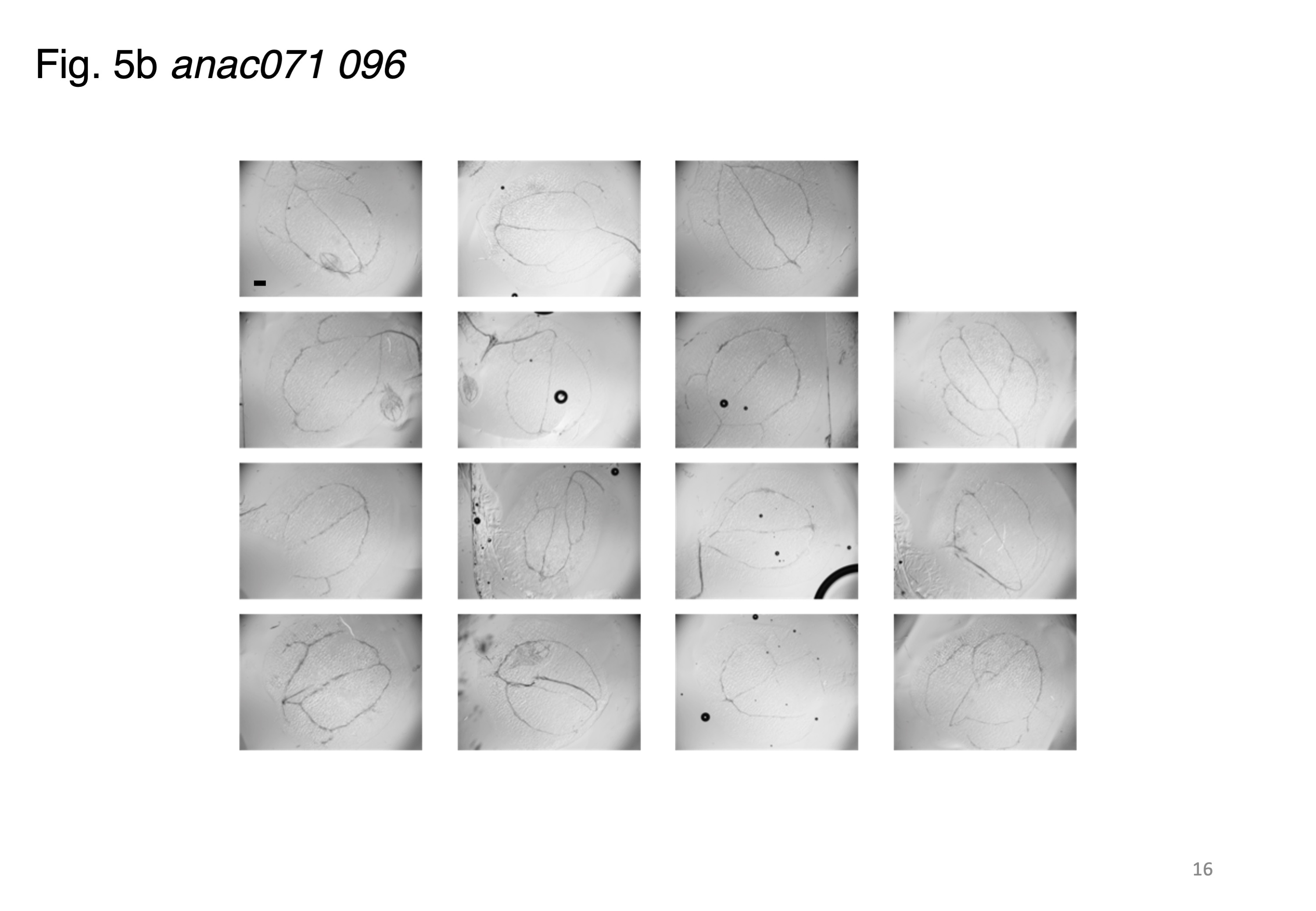

Supplement: Supplementary file 5 — Supplementary Data 1 [file 42003_2021_1895_MOESM5_ESM.zip › SupplementaryDataset1/16.jpeg]

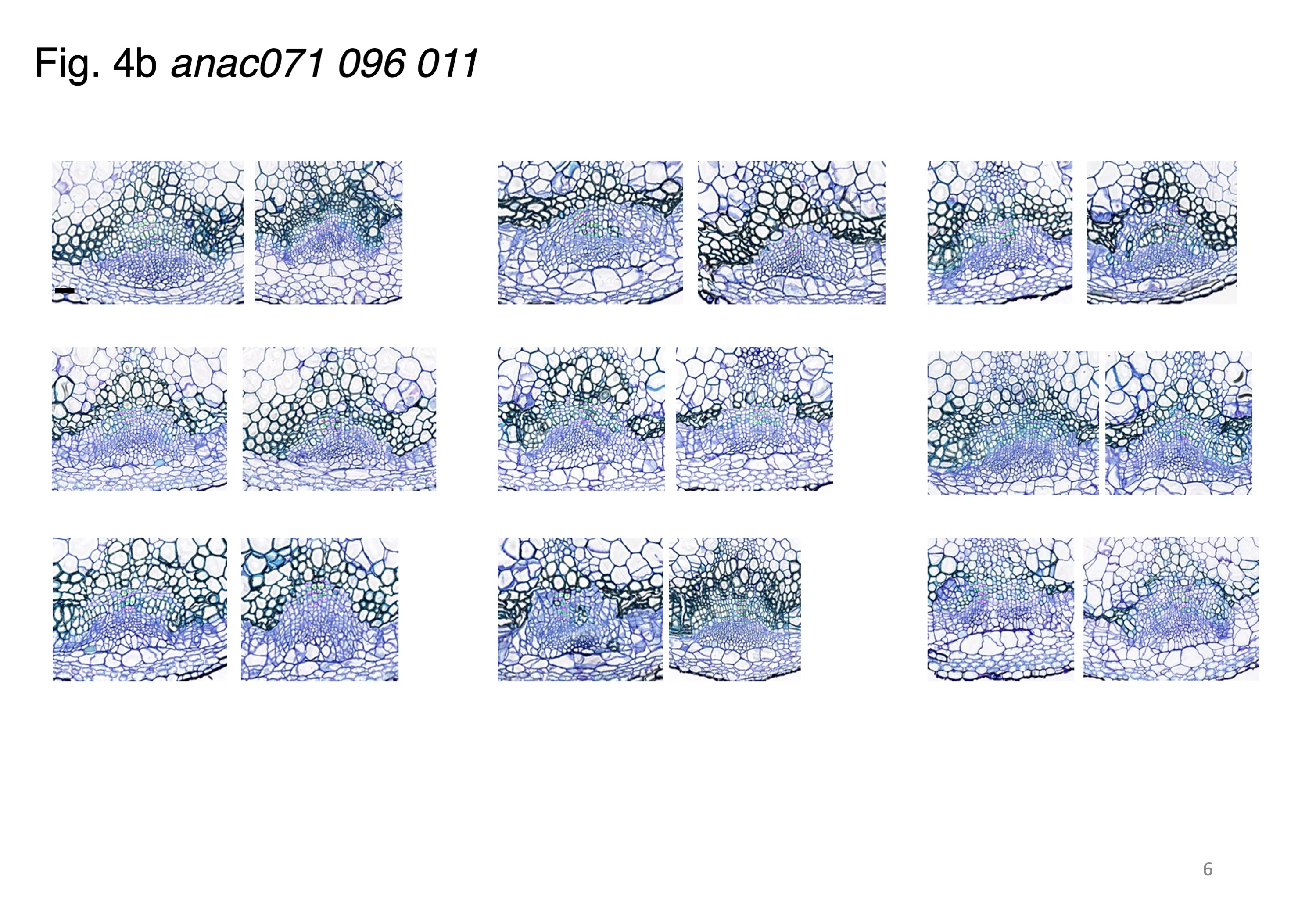

Supplement: Supplementary file 5 — Supplementary Data 1 [file 42003_2021_1895_MOESM5_ESM.zip › SupplementaryDataset1/6.jpeg]

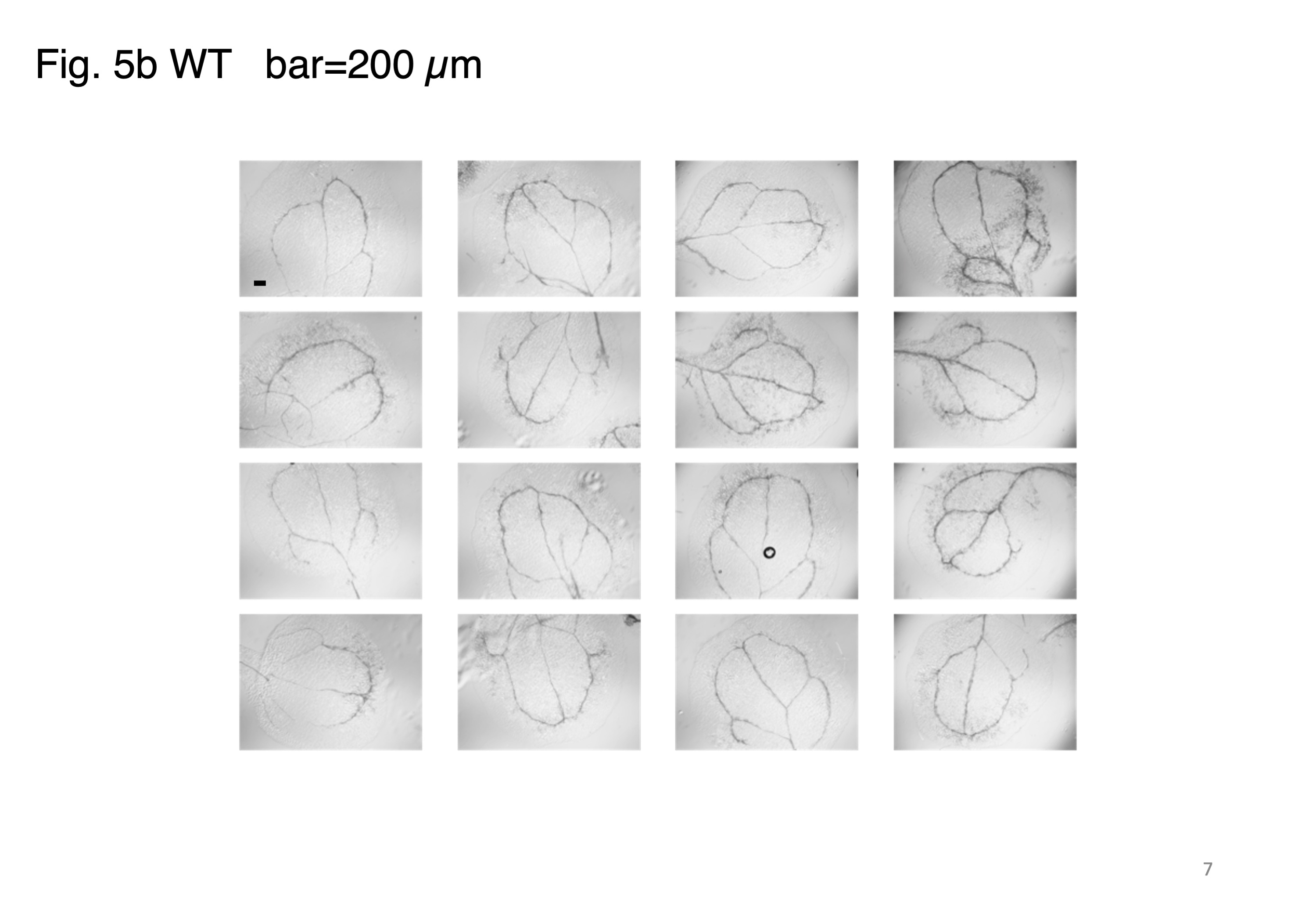

Supplement: Supplementary file 5 — Supplementary Data 1 [file 42003_2021_1895_MOESM5_ESM.zip › SupplementaryDataset1/7.jpeg]

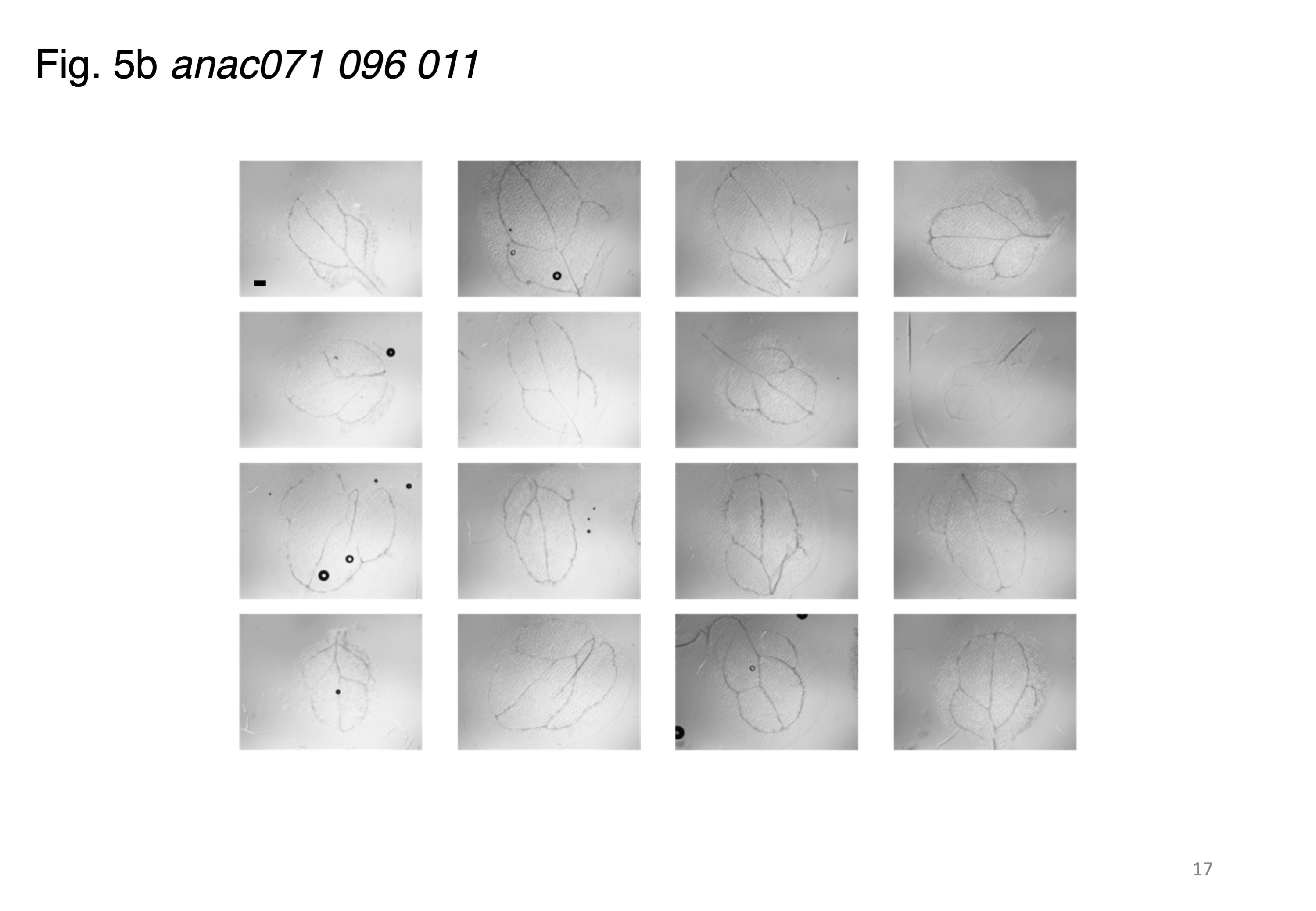

Supplement: Supplementary file 5 — Supplementary Data 1 [file 42003_2021_1895_MOESM5_ESM.zip › SupplementaryDataset1/17.jpeg]

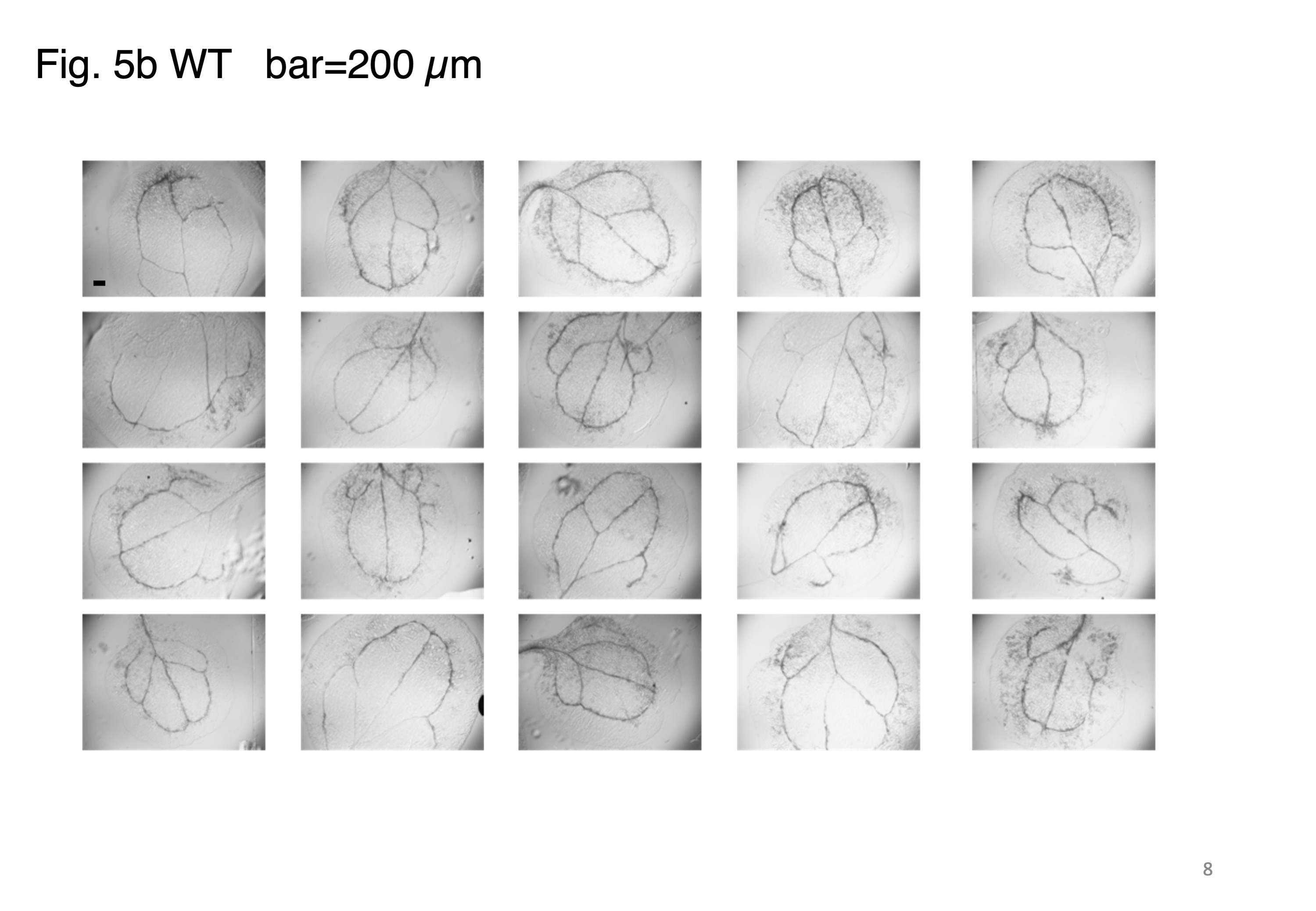

Supplement: Supplementary file 5 — Supplementary Data 1 [file 42003_2021_1895_MOESM5_ESM.zip › SupplementaryDataset1/8.jpeg]

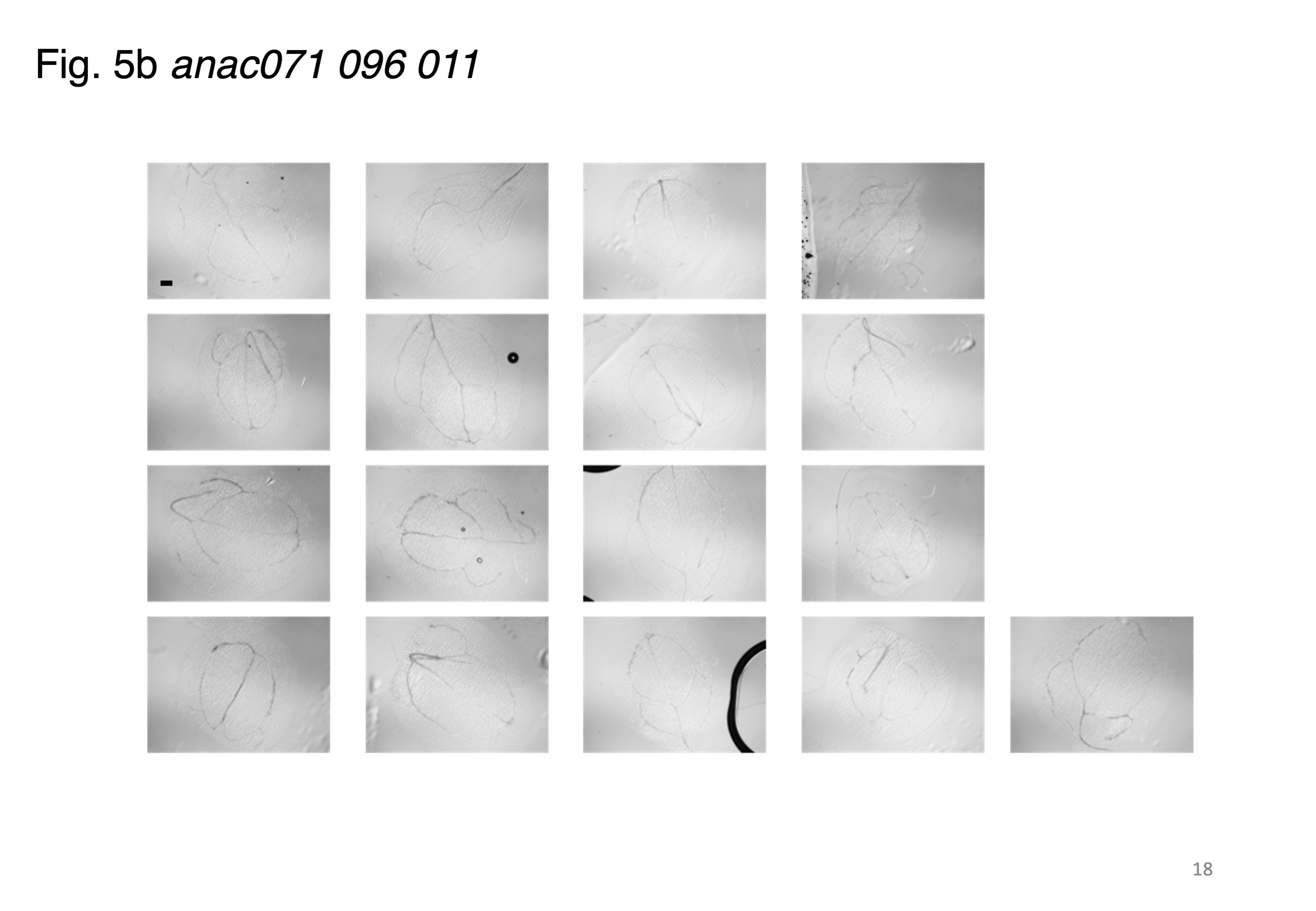

Supplement: Supplementary file 5 — Supplementary Data 1 [file 42003_2021_1895_MOESM5_ESM.zip › SupplementaryDataset1/18.jpeg]

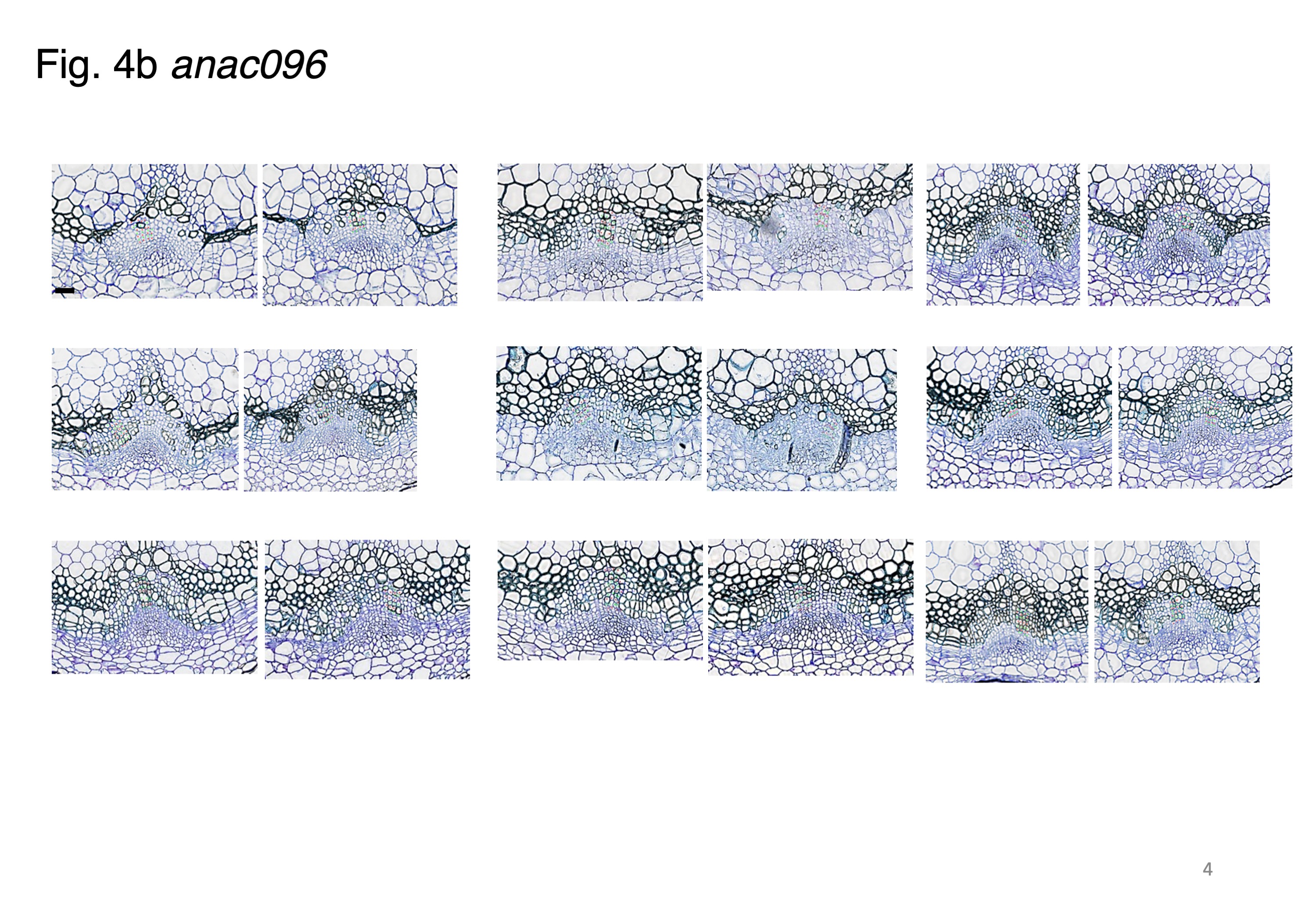

Supplement: Supplementary file 5 — Supplementary Data 1 [file 42003_2021_1895_MOESM5_ESM.zip › SupplementaryDataset1/4.jpeg]

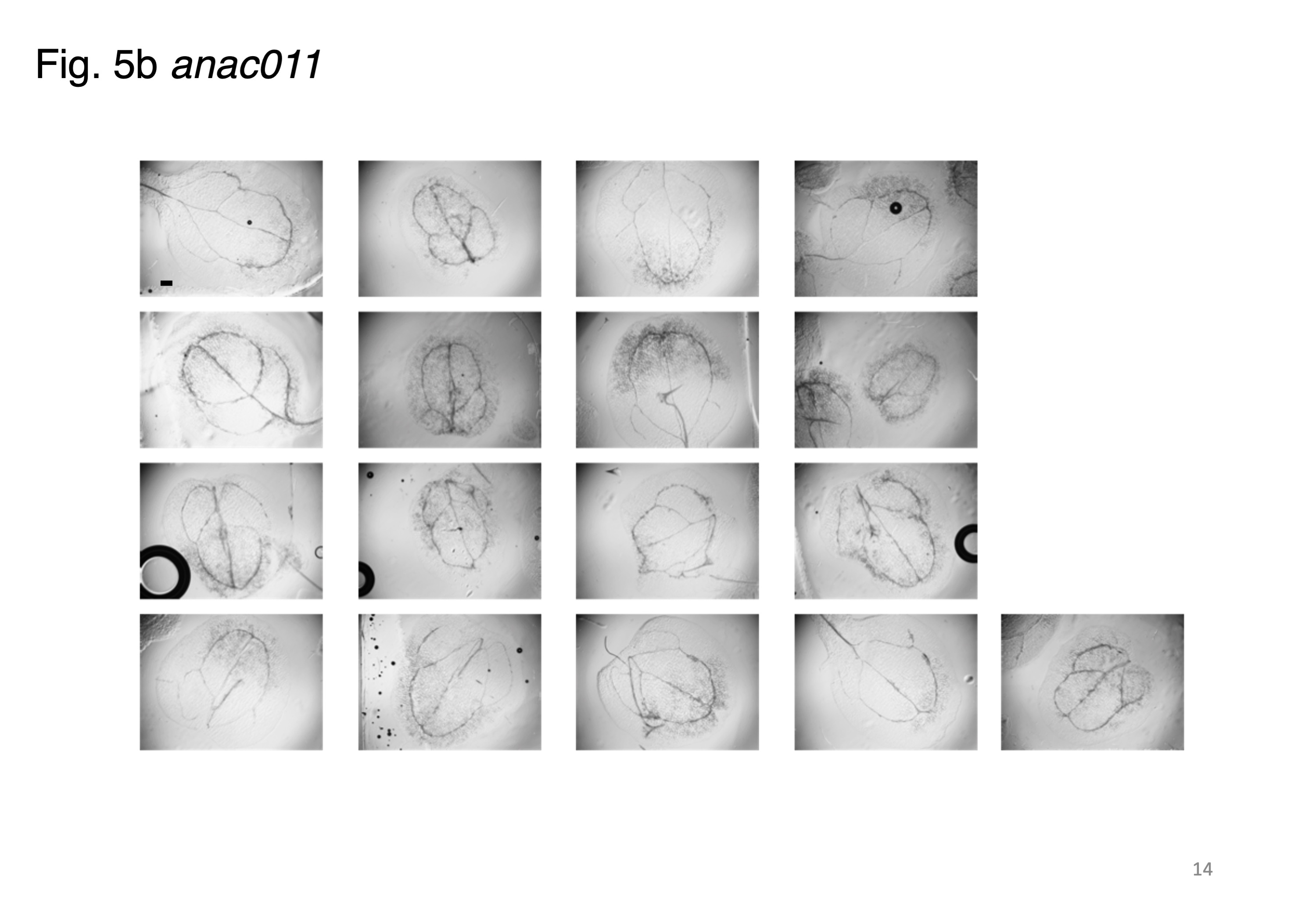

Supplement: Supplementary file 5 — Supplementary Data 1 [file 42003_2021_1895_MOESM5_ESM.zip › SupplementaryDataset1/14.jpeg]

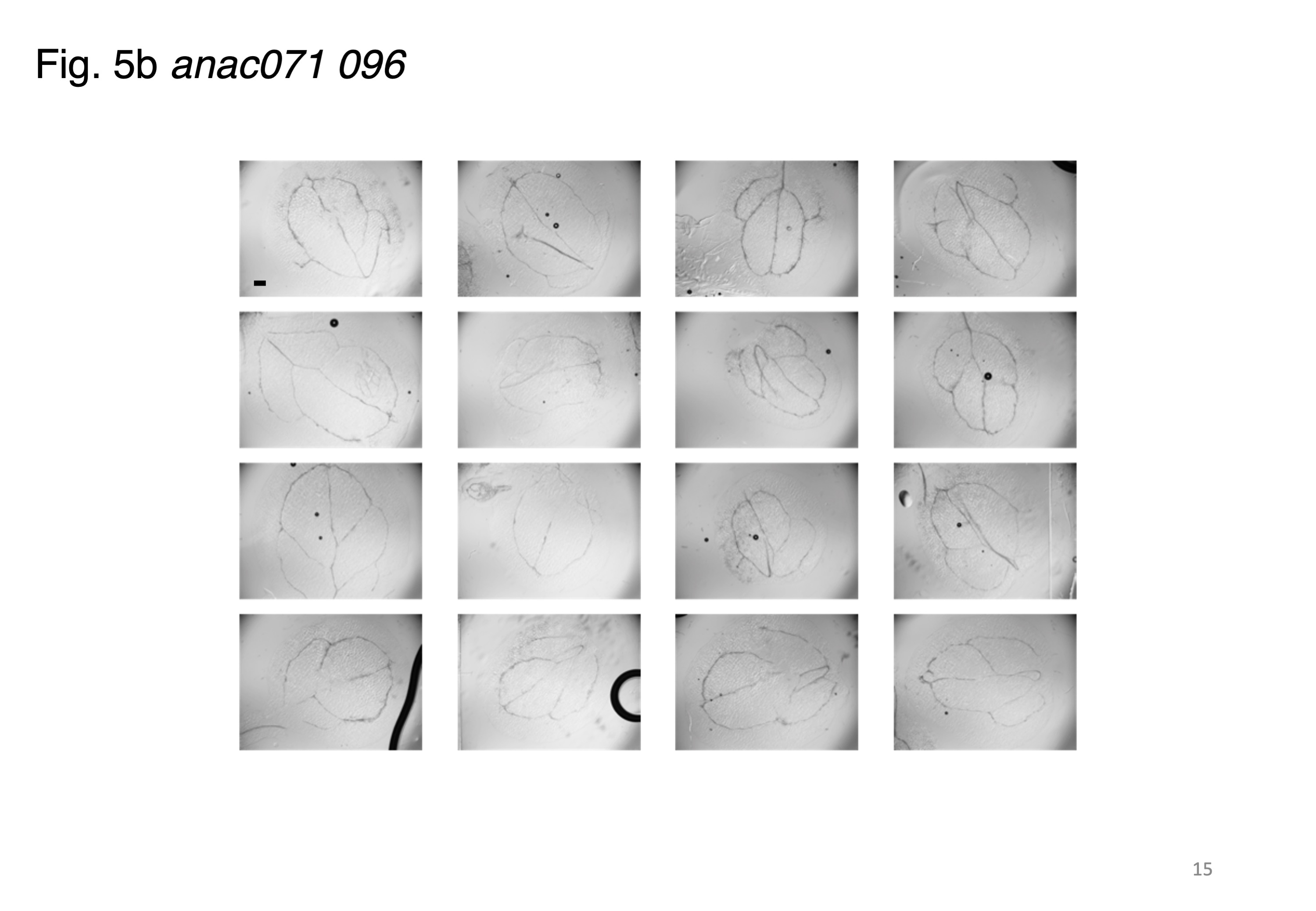

Supplement: Supplementary file 5 — Supplementary Data 1 [file 42003_2021_1895_MOESM5_ESM.zip › SupplementaryDataset1/15.jpeg]

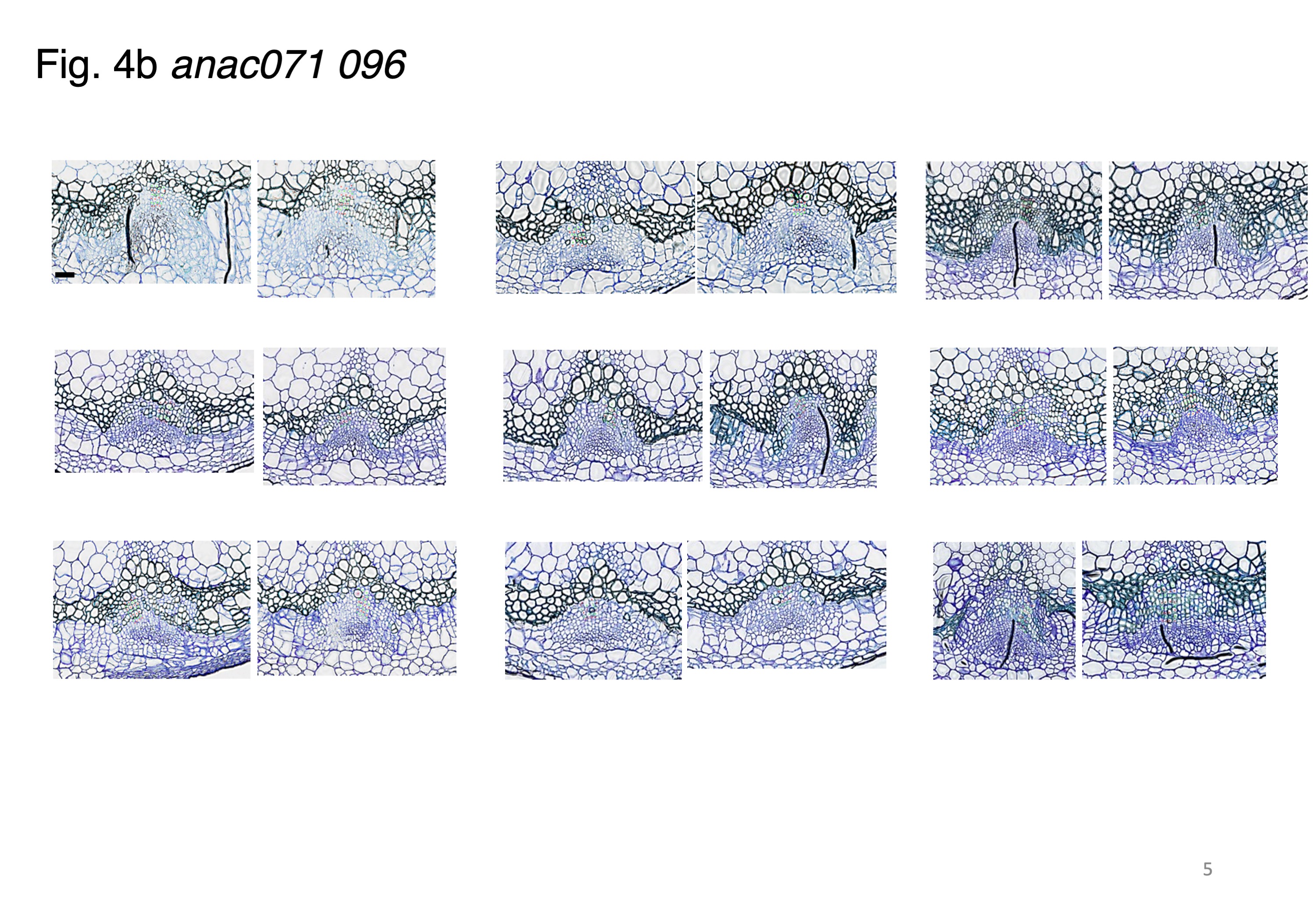

Supplement: Supplementary file 5 — Supplementary Data 1 [file 42003_2021_1895_MOESM5_ESM.zip › SupplementaryDataset1/5.jpeg]

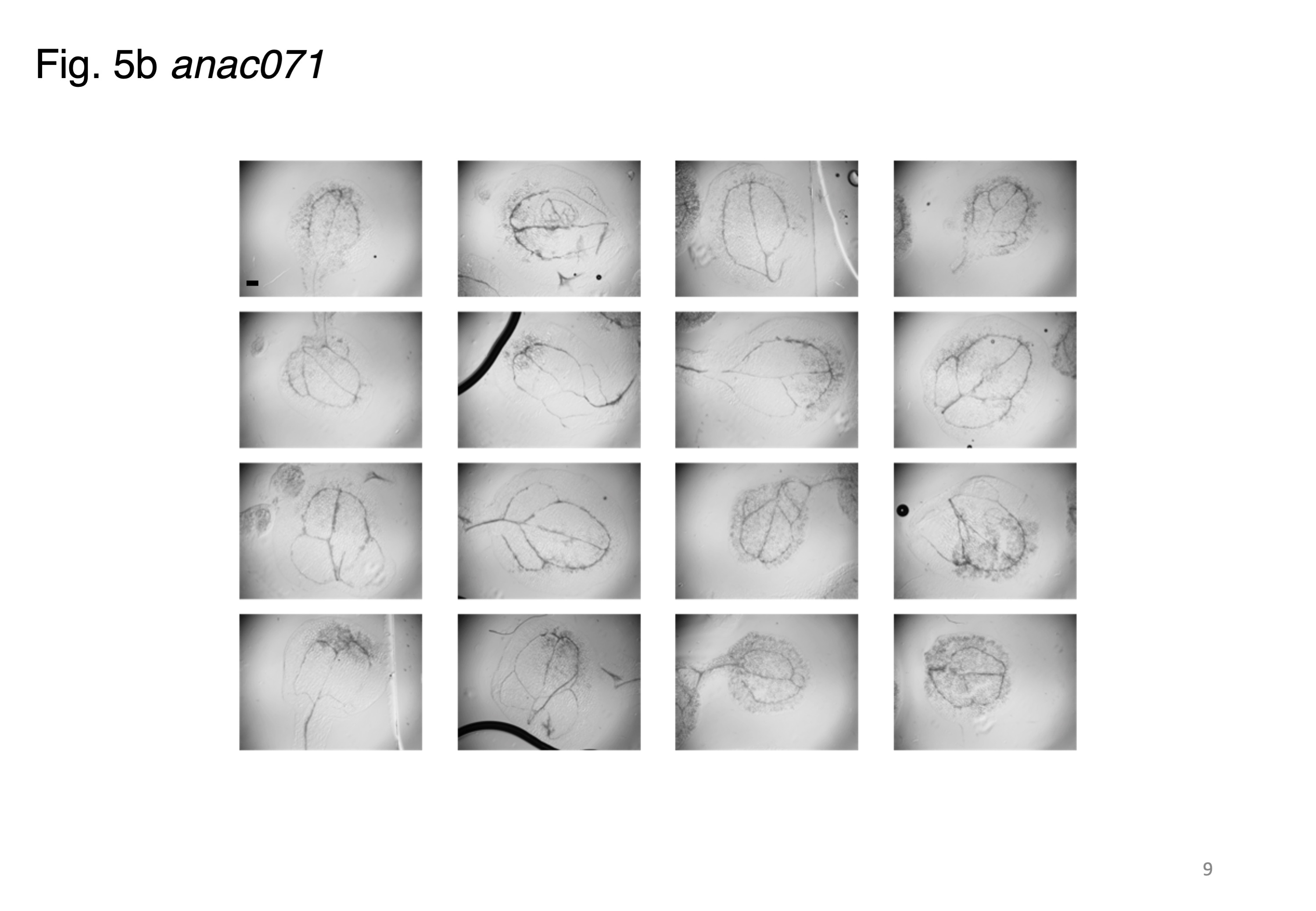

Supplement: Supplementary file 5 — Supplementary Data 1 [file 42003_2021_1895_MOESM5_ESM.zip › SupplementaryDataset1/9.jpeg]

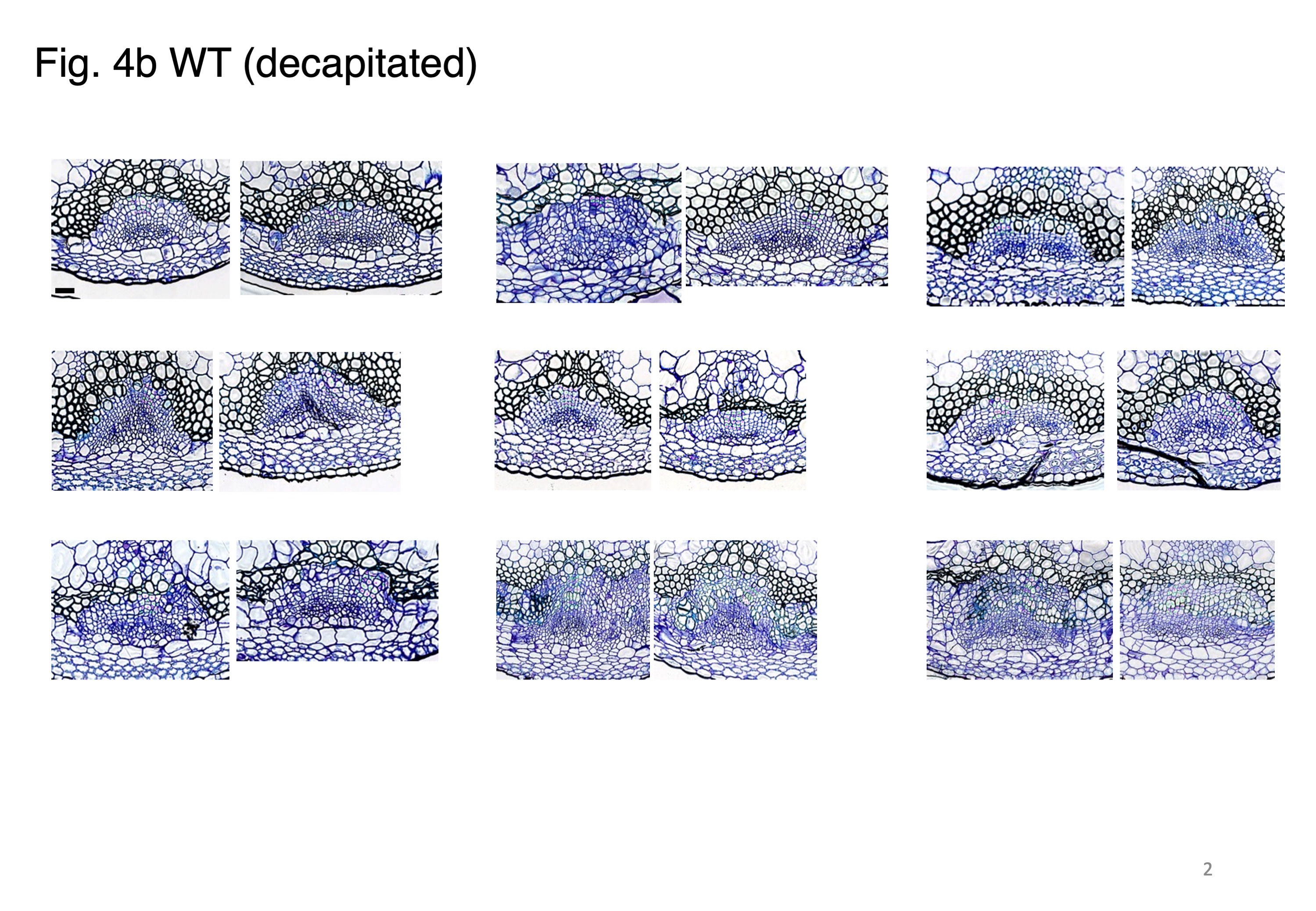

Supplement: Supplementary file 5 — Supplementary Data 1 [file 42003_2021_1895_MOESM5_ESM.zip › SupplementaryDataset1/2.jpeg]

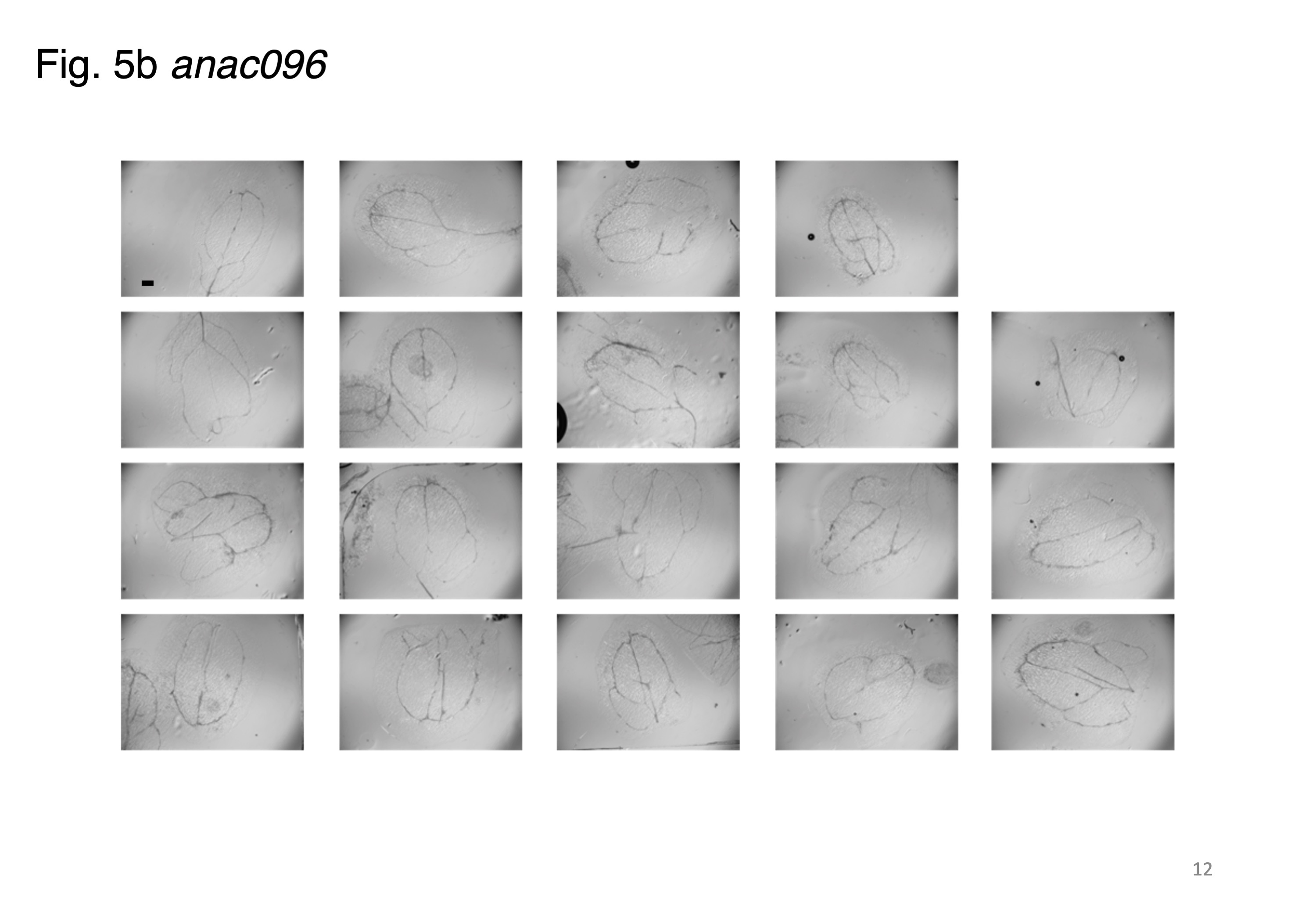

Supplement: Supplementary file 5 — Supplementary Data 1 [file 42003_2021_1895_MOESM5_ESM.zip › SupplementaryDataset1/12.jpeg]

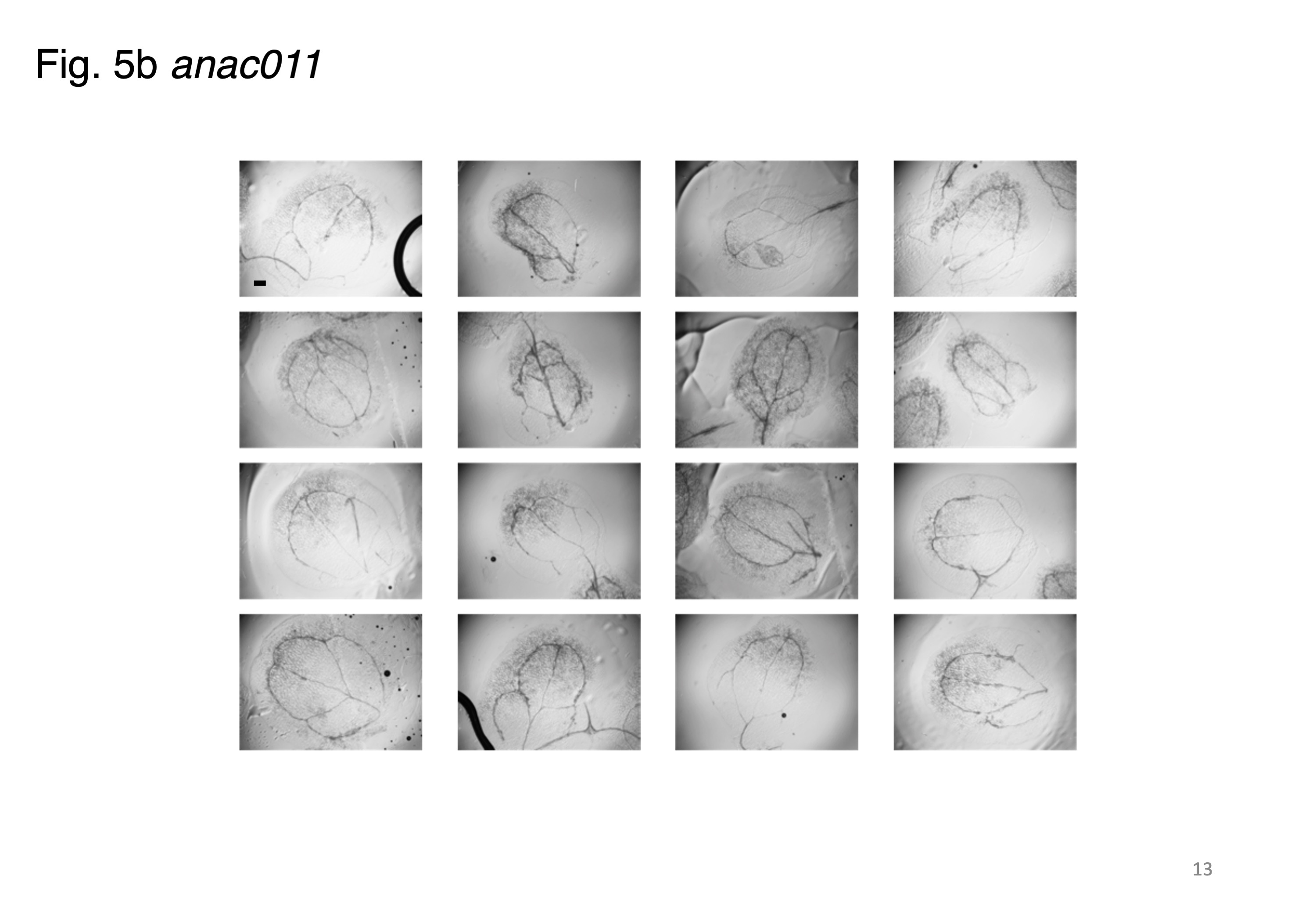

Supplement: Supplementary file 5 — Supplementary Data 1 [file 42003_2021_1895_MOESM5_ESM.zip › SupplementaryDataset1/13.jpeg]

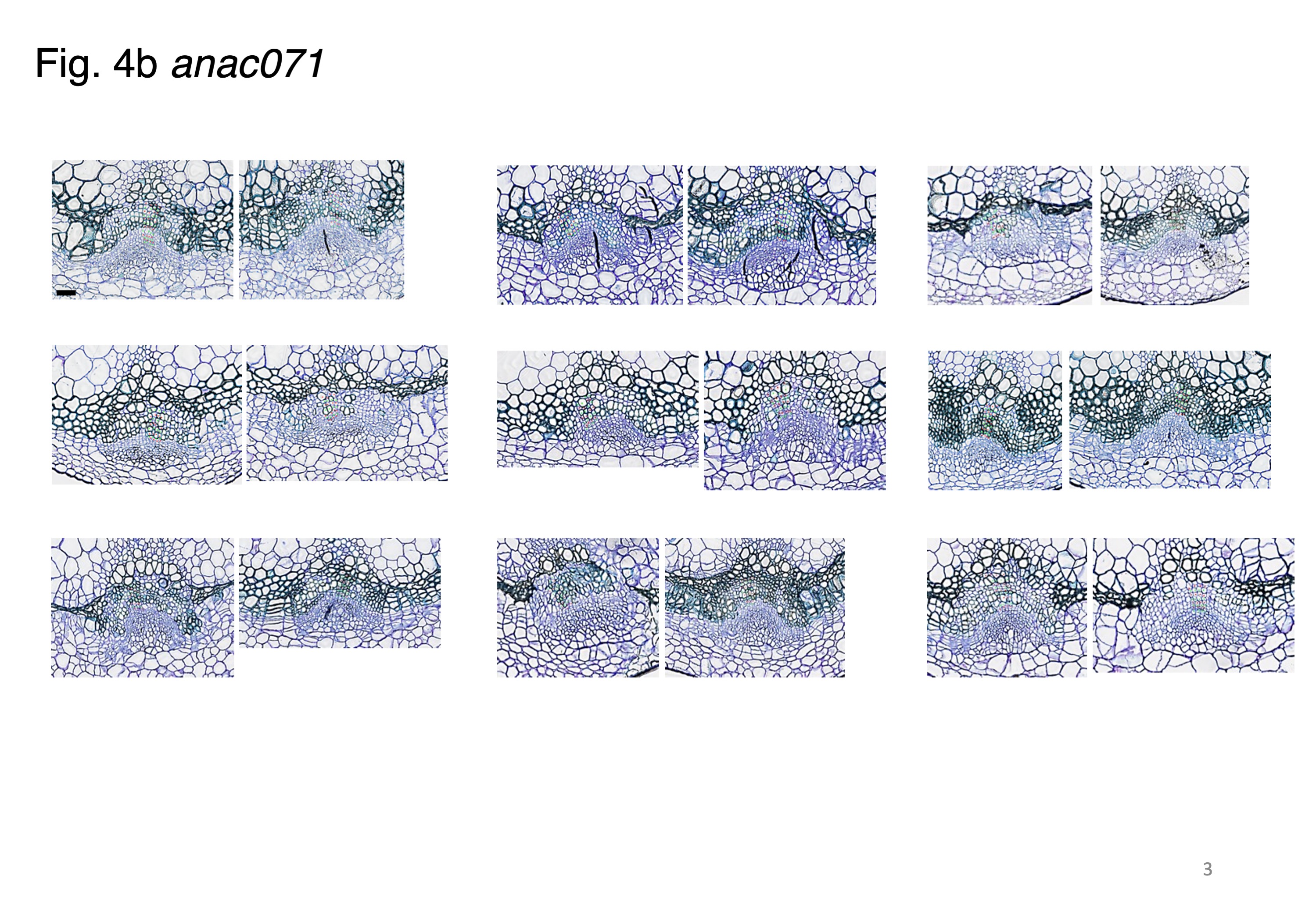

Supplement: Supplementary file 5 — Supplementary Data 1 [file 42003_2021_1895_MOESM5_ESM.zip › SupplementaryDataset1/3.jpeg]
