# Supplementary material for: Wound-inducible ANAC071 and ANAC096 transcription factors promote cambial cell formation in incised Arabidopsis flowering stems
Source: Commun Biol. 2021 Mar 19;4:369. doi: 10.1038/s42003-021-01895-8 (PMC7979829; doi:10.1038/s42003-021-01895-8)
Supplement: Supplementary file 7 — Reporting Summary [file 42003_2021_1895_MOESM7_ESM.pdf]

nature research

Corresponding author(s): Masashi Asahina
Last updated by author(s): 2021/02/04

Reporting Summary

Nature Research wishes to improve the reproducibility of the work that we publish. This form provides structure for consistency and transparency in reporting. For further information on Nature Research policies, see our Editorial Policies and the Editorial Policy Checklist.

Statistics

For all statistical analyses, confirm that the following items are present in the figure legend, table legend, main text, or Methods section.

n/a	Confirmed	
☐	☒	The exact sample size (*n*) for each experimental group/condition, given as a discrete number and unit of measurement
☐	☒	A statement on whether measurements were taken from distinct samples or whether the same sample was measured repeatedly
☐	☒	The statistical test(s) used AND whether they are one- or two-sided<br>*Only common tests should be described solely by name; describe more complex techniques in the Methods section.*
☐	☒	A description of all covariates tested
☐	☒	A description of any assumptions or corrections, such as tests of normality and adjustment for multiple comparisons
☐	☒	A full description of the statistical parameters including central tendency (e.g. means) or other basic estimates (e.g. regression coefficient) AND variation (e.g. standard deviation) or associated estimates of uncertainty (e.g. confidence intervals)
☒	☐	For null hypothesis testing, the test statistic (e.g. *F*, *t*, *r*) with confidence intervals, effect sizes, degrees of freedom and *P* value noted<br>*Give P values as exact values whenever suitable.*
☒	☐	For Bayesian analysis, information on the choice of priors and Markov chain Monte Carlo settings
☒	☐	For hierarchical and complex designs, identification of the appropriate level for tests and full reporting of outcomes
☒	☐	Estimates of effect sizes (e.g. Cohen's *d*, Pearson's *r*), indicating how they were calculated

Our web collection on statistics for biologists contains articles on many of the points above.

Software and code

Policy information about availability of computer code

Data collection	Confocal image acquisition was performed by Leica SP8 microscope using LAS X 3.1.5.16308.<br>Light microscopy image was taken by Leica M165FC stereomicroscope with a MC120 HD camera using LAS suite 4.12.0, or APERIO CS2 using Scanner Console 102.0.74.<br>Fluorescent optical image were obtained by Olympus BX53 microscope with a DP73 camera using Olympus cellSens Standard 1.13.
Data analysis	Images of serial sections were aligned using Fiji/TrakEM2 software (https://imagej.net/TrakEM2), and the tissue image was painted with false colors. 3D image was generated using the ImageJ 3D viewer program (https://imagej.net).<br>Multiple sequence alignment was conducted using the online version of MAFFT with the L-INS-I option (https://mafft.cbrc.jp/alignment/server/), and MEGA version 7.0 software (https://www.megasoftware.net) were used to draw the phylogenetic tree.

For manuscripts utilizing custom algorithms or software that are central to the research but not yet described in published literature, software must be made available to editors and reviewers. We strongly encourage code deposition in a community repository (e.g. GitHub). See the Nature Research guidelines for submitting code & software for further information.

Data

Policy information about availability of data

All manuscripts must include a data availability statement. This statement should provide the following information, where applicable:

- Accession codes, unique identifiers, or web links for publicly available datasets
- A list of figures that have associated raw data
- A description of any restrictions on data availability

All data and genetic material used for this paper are available from the corresponding author upon request.

Field-specific reporting

Please select the one below that is the best fit for your research. If you are not sure, read the appropriate sections before making your selection.

[x] Life sciences [ ] Behavioural & social sciences [ ] Ecological, evolutionary & environmental sciences

For a reference copy of the document with all sections, see nature.com/documents/nr-reporting-summary-flat.pdf

Life sciences study design

All studies must disclose on these points even when the disclosure is negative.

Sample size	No statistical methods were used to determine sample size. Sample sizes were determined based on previous experience and standards in the field. Sample size for each experiments are shown in the figure legend.
Data exclusions	No data were excluded from the analysis. Light microscopy experiments were done in multiple times with essentially the same result, and representative results are shown (Fig.1, Fig.3, Fig.4a, Fig.5a, Fig.6a,6g, Fig.7e-g, and Supplementary Figures).
Replication	All experiments were repeated at least three times using independently grown plant materials, with essentially the same result.
Randomization	Not applicable.
Blinding	Blinding was not relevant to this study.

Reporting for specific materials, systems and methods

We require information from authors about some types of materials, experimental systems and methods used in many studies. Here, indicate whether each material, system or method listed is relevant to your study. If you are not sure if a list item applies to your research, read the appropriate section before selecting a response.

Materials & experimental systems

n/a	Involved in the study
[x]	[ ] Antibodies
[x]	[ ] Eukaryotic cell lines
[x]	[ ] Palaeontology and archaeology
[x]	[ ] Animals and other organisms
[x]	[ ] Human research participants
[x]	[ ] Clinical data
[x]	[ ] Dual use research of concern

Methods

n/a	Involved in the study
[x]	[ ] ChIP-seq
[x]	[ ] Flow cytometry
[x]	[ ] MRI-based neuroimaging
